# Annotation of CD8^+^ T-cell function via ICAM-1 imaging identifies FAK inhibition as an adjuvant to augment the antitumor immunity of radiotherapy

**DOI:** 10.7150/thno.90709

**Published:** 2024-01-01

**Authors:** Ting Zhang, Yining Zhang, Yang Zhao, Rui Song, Yanpu Wang, Kui Li, Haoyi Zhou, Feng Wang, Shixin Zhou, Meixin Zhao, Hua Zhu, Weifang Zhang, Zhi Yang, Zhaofei Liu

**Affiliations:** 1 Department of Radiation Medicine, School of Basic Medical Sciences, Peking University Health Science Center, Beijing 100191, China.; 2 Key Laboratory of Carcinogenesis and Translational Research (Ministry of Education/Beijing), Key Laboratory for Research and Evaluation of Radiopharmaceuticals (National Medical Products Administration), Department of Nuclear Medicine, Peking University Cancer Hospital and Institute, Beijing 100142, China.; 3 Department of Cell Biology, School of Basic Medical Sciences, Peking University Health Science Center, Beijing 100191, China.; 4 Department of Nuclear Medicine, Peking University Third Hospital, Beijing 100191, China.; 5 State Key Laboratory of Vascular Homeostasis and Remodeling, Peking University, Beijing 100191, China.; 6 Peking University-Yunnan Baiyao International Medical Research Center, Beijing 100191, China.

**Keywords:** molecular imaging, positron emission tomography, radiotherapy, ICAM-1, focal adhesion kinase

## Abstract

**Background:** Radiotherapy (RT) may trigger systemic antitumor immunity, manifesting as regression of non-irradiated lesions (abscopal effect). Intracellular adhesion molecule-1 (ICAM-1) is a key molecule involved in the abscopal effect of RT. However, the specific function of ICAM-1 in CD8^+^ T cells during antitumor immune responses remains unclear. Herein, we investigated whether noninvasive imaging of ICAM-1 can be used to annotate CD8^+^ T-cell function, thereby better selecting combinational therapy to enhance the antitumor immunity induced by RT.

**Methods:** Using knockout mouse models, we investigated the role of ICAM-1 expressed on CD8^+^ T cells in the antitumor immunity of RT and conducted drug screening guided by ICAM-1-targeted noninvasive imaging.

**Results:** The systemic antitumor effect of RT relies on the expression of ICAM-1 on CD8^+^ T cells. ICAM-1 expression is essential for CD8^+^ T-cell activation, proliferation, and effector function. Noninvasive annotation of the proliferation and effector function of CD8^+^ T cells by ICAM-1-targeted imaging identified VS-6063, a focal adhesion kinase inhibitor, as a new adjuvant to augment systemic antitumor immunity of RT in an immunologically “cold” tumor model. Mechanistically, VS-6063 overcomes the physical barriers in tumors and promotes the migration and infiltration of CD8^+^ T cells primed by RT into distant tumors.

**Conclusion:** Our findings highlight that molecular imaging of ICAM-1 levels provides a dynamic readout of the proliferation and effector function of tumor-infiltrating CD8^+^ T cells, which facilitates the high-throughput exploitation of new combinational drugs to maximize the systemic antitumor effect of RT.

## Introduction

Radiotherapy (RT) is the mainstay of cancer treatment, with over half of all patients with cancer receiving RT as part of their treatment regimens 1,2. Besides its commonly recognized local effect of causing direct DNA damage to irradiated tumor cells, RT may trigger systemic antitumor immunity, manifesting as regression of lesions in the non-irradiated field (abscopal effect) 3,4. Harnessing the power of the systemic antitumor effect of RT may facilitate effective treatment of primary tumors and eliminate metastatic lesions or lesions that cannot be directly irradiated. However, the occurrence of the systemic antitumor effect of RT in patients is extremely rare 5,6. Combinations of RT with immunotherapies, represented by immune checkpoint inhibitors 7-10, have been extensively investigated in clinical trials to augment the systemic antitumor effects of RT. However, these trials are typically guided by empirical choices, with response rates varying greatly due to several factors, including radiation dose, fractionation, and timing, as well as the selected immunotherapy. Moreover, treatment with immune checkpoint inhibitors may cause immunotherapy-related adverse events, including highly lethal myocarditis 11. Therefore, the development of a reliable approach to guide the rational design of RT combinations is urgently required.

Molecular imaging approaches, such as positron emission tomography (PET) and near-infrared fluorescence (NIRF) imaging, have facilitated the characterization and quantification of biological molecules in a noninvasive and quantitative manner 12,13. Although the fundamental mechanism underlying the systemic antitumor effect of RT remains nebulous, it may involve the induction of immunogenic cell death to stimulate antitumor CD8^+^ T-cell responses. Therefore, molecular imaging of the proliferation and effector function of CD8^+^ T cells may guide the RT combinational regimens and assist in the selection of new drugs in RT combinations.

Recent studies have demonstrated that PET imaging of CD8^+^ T cells using radiolabeled anti-CD8 antibodies can noninvasively characterize T-cell infiltration during immunotherapy in clinical settings 14,15, which helps to characterize T-cell-based antitumor immunity. However, characterization of the total population of CD8^+^ T cells using CD8-targeted PET imaging is not a reliable predictor of the antitumor immunity clinical benefit as this relies on the activation and effector function of CD8^+^ T cells rather than only total CD8^+^ populations 16,17. Accordingly, PET of T-cell effector function markers, such as granzyme B, offers new strategies for characterizing functional CD8^+^ T-cell populations 18,19. Our previous work showed that granzyme B-targeted PET using the radiotracer^ 68^Ga-grazytracer could noninvasively characterize CD8^+^ T-cell effector function and monitor early tumor responses to immunotherapy in patients 18. However, given that granzyme B is a secreted protein, longitudinal PET is required to accurately capture its secretion window and provide data on T-cell effector function. Therefore, further studies are needed to develop new biomarker-targeted imaging approaches that can annotate the activation and effector function of CD8^+^ T cells to facilitate RT combinational therapies.

We recently reported substantial upregulation of intracellular adhesion molecule-1 (ICAM-1) in non-irradiated tumors responsive to RT and identified ICAM-1 as a key molecule involved in the abscopal effect of RT 20. ICAM-1, a transmembrane glycoprotein, is expressed on leukocytes and endothelial cells, of which, the lymphocyte function-associated antigen-1 (LFA-1) is the primary ligand/receptor of ICAM-1. The interaction between ICAM-1 and LFA-1 mediates various physiological processes that respond to immunological and inflammatory reactions, including the transmembrane migration of lymphocytes, the formation of immune synapses, and antigen recognition and targeted killing 21,22. Besides, researches have shown that ICAM-1 is also involved in cluster formation and differentiation of T cells 23,24. Given the important role of T-cell immunity in systemic antitumor effect 3, we hypothesize that ICAM-1 is involved in RT-induced responses of T cells. However, the specific function of ICAM-1 in CD8^+^ T cells during antitumor immune responses of RT remains unclear.

In this study, we showed that ICAM-1 deficiency impairs the ability of RT to induce the systemic antitumor effect by disrupting the antitumor immunity of CD8^+^ T cells and demonstrated the role of ICAM-1 in the proliferative capacity and cytotoxic function of CD8^+^ T cells. Therefore, we developed a noninvasive imaging strategy using ICAM-1-targeted NIRF imaging or more clinically relevant PET to guide drug screening, and revealed that focal adhesion kinase (FAK) inhibition could markedly enhance the systemic antitumor effect of RT. As several FAK inhibitors are currently under investigation in clinical trials 25, RT in combination with FAK inhibition is a promising approach to effectively treat primary tumors and disseminated lesions in clinical settings.

## Results

### ICAM-1 deficiency diminishes RT-induced systemic antitumor effects

**Figure 1:**
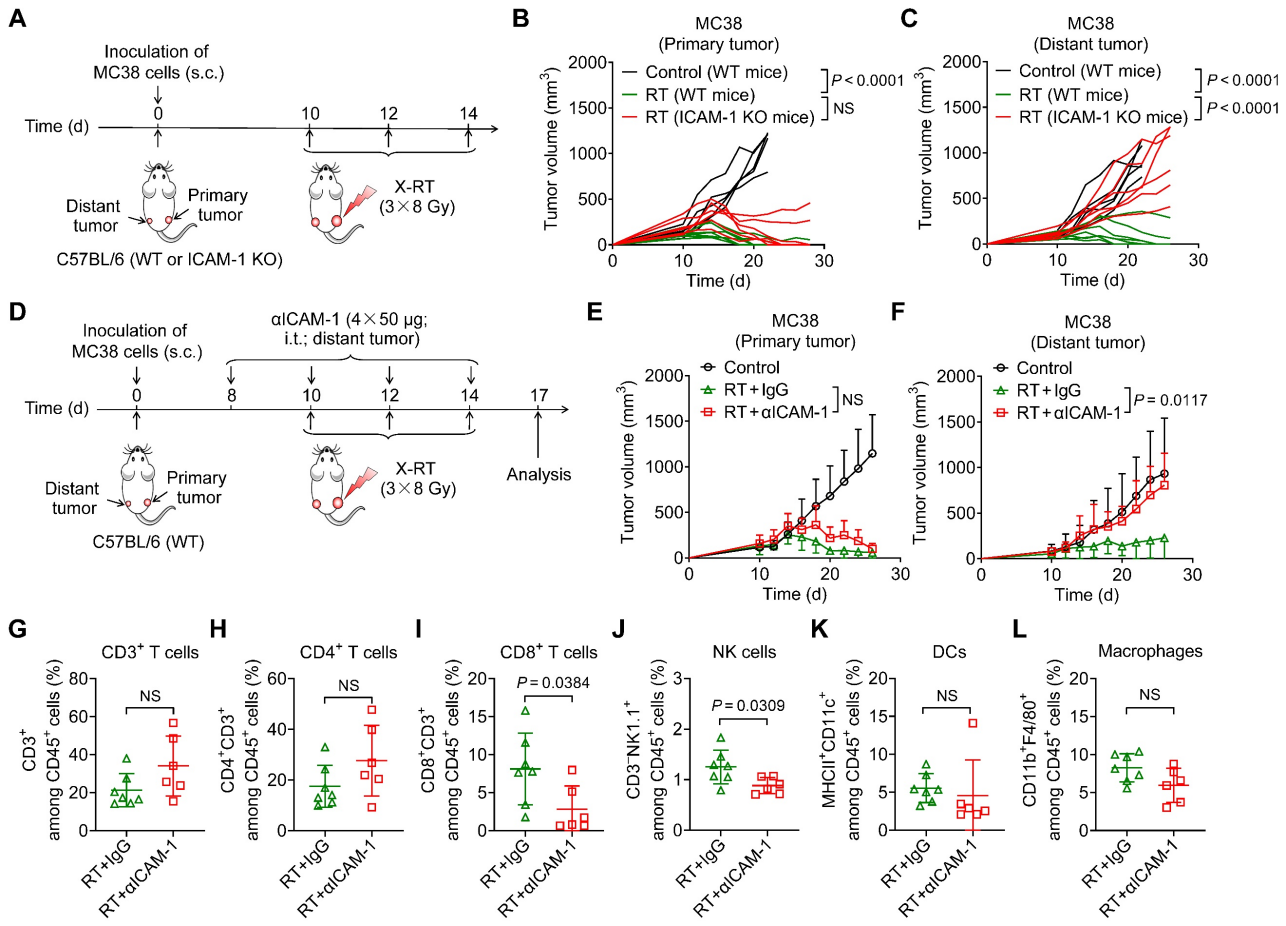
ICAM-1 deficiency impairs the systemic antitumor effect of RT. (A) Schedule of X-ray RT in the WT or ICAM-1 KO C57BL/6 mice bearing bilateral MC38 tumors. (B, C) Individual tumor growth curves of the primary tumors (B) and distant tumors (C) of MC38 tumor-bearing mice after RT as illustrated in (A) (n = 5-7 per group). (D) Schedule of RT in combination with ICAM-1 blocking with an anti-ICAM-1 antibody (αICAM-1) in the C57BL/6 WT mice bearing bilateral MC38 tumors. (E, F) Average tumor growth curves of primary tumors (E) and distant tumors (F) in the MC38 tumor-bearing mice after indicated treatments (n = 4-7 per group). (G-I) Frequencies of CD3^+^ T cells (G), CD4^+^ T cells (H), and CD8^+^ T cells (I) among CD45^+^ cells from distant tumors harvested from WT C57BL/6 mice after indicated treatments as described in (D) (n = 6-7 per group). (J-L) Frequencies of NK cells (J), DCs (K), and macrophages (L) among CD45^+^ cells from distant tumors harvested from WT C57BL/6 mice after indicated treatments as described in (D) (n = 6-7 per group). Data are represented as mean ± SD. *P* values were determined using unpaired Student's *t*-test (B, C, G-L), or two-way ANOVA with Tukey's multiple-comparisons test (E, F); NS, not significant (*P* > 0.05).

To investigate the role of ICAM-1 in the systemic antitumor effect of RT, we used a highly immunogenic MC38 tumor model 26 by subcutaneously implanting tumor cells into the right hind flank (primary; irradiated tumor) and left hind flank (distant; non-irradiated tumor) of wild-type (WT) or ICAM-1 knockout (ICAM-1 KO) C57BL/6 mice (**Figure 1A**). In the MC38 WT C57BL/6 mouse model, RT may greatly induce systemic antitumor immunity 27,28. A total of 24 Gy in three fractions (3 × 8 Gy) has shown a better performance in promoting RT-induced tumor immunogenicity 29; herein, it effectively ablated primary tumors and led to relatively complete regression of the non-irradiated distant tumors in the MC38 WT C57BL/6 tumor model (**Figure 1B**, **C**).

We further investigated the effect of ICAM-1 deficiency on the systemic antitumor effect of RT using ICAM-1 KO C57BL/6 mice (**Figure 1A**), which allowed for genetic ablation of ICAM-1 throughout the RT procedure. ICAM-1 KO in C57BL/6 mice did not affect the growth of MC38 tumors (**Figure S1A**,** B**). Although RT almost fully inhibited the growth of primary MC38 tumors in ICAM-1 KO mice, genetic ablation of ICAM-1 nearly abrogated the efficacy of RT in distant tumors (**Figure 1B**, **C**). These results were also confirmed in the ICAM-1 WT or KO mice in another syngeneic B16-F10 murine melanoma model (**Figure S2A**-**C**). This demonstrates the pivotal role of ICAM-1 in the systemic antitumor effect of RT.

To confirm these findings, we performed ICAM-1 blockade via intratumoral injection of an anti-ICAM-1 antibody into distant tumors (**Figure 1D**). Compared with injection of phosphate-buffered saline (PBS) control, injection of the anti-ICAM-1 antibody alone did not affect tumor growth (**Figure S3A**,** B**) and exerted limited effects on the tumor-infiltrating immune cell population (**Figure S3C**-**H**). However, combination with RT, although* in vivo* ICAM-1 blocking in distant tumors had limited effects on primary tumor growth, significantly diminished the RT-induced systemic antitumor effect in distant tumors compared with that in the RT plus isotype-matched IgG group (*P* <0.05; **Figure 1E**,** F** and **Figure S4A**,** B**).

To understand the mechanisms underlying the abrogated systemic antitumor response mediated by ICAM-1 deficiency *in vivo*, we analyzed the immune cell populations present in distant tumors. Flow cytometric analysis showed that ICAM-1 blockade significantly reduced CD8^+^ T-cell infiltration (*P* <0.05) in distant tumors without significantly impacting the CD3^+^ and CD4^+^ T-cell frequencies (**Figure 1G**-**I**). In addition, blocking ICAM-1 reduced the frequency of natural killer (NK) cells (*P* <0.05) without affecting dendritic cells (DCs) and macrophages in distant tumors (**Figure 1J**-**L**). Taken together, our results suggest that ICAM-1 deficiency impairs the infiltration of immune cells, including CD8^+^ T cells and NK cells, into distant tumors outside the irradiated field, thereby diminishing the systemic antitumor effect of RT.

### Blocking T-cell infiltration elicits the same effect as ICAM-1 deficiency in distant tumors

**Figure 2:**
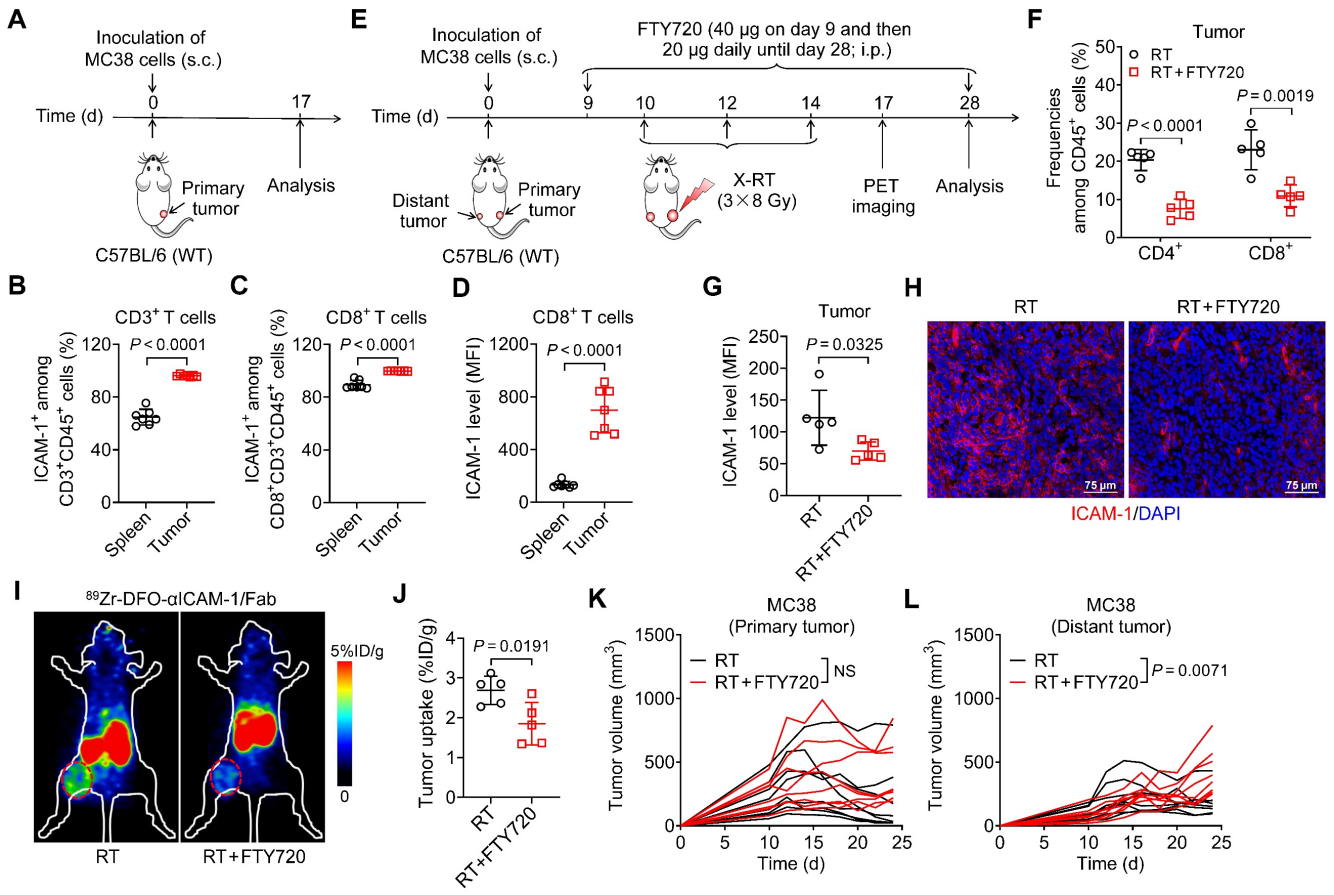
Blocking T-cell migration to tumors with FTY720 eliminates ICAM-1 expression in tumors and abrogates the systemic antitumor effect of RT. (A) Schedule of the flow cytometric analysis of WT C57BL/6 mice bearing MC38 tumors. (B, C) Frequencies of ICAM-1^+^ cells among CD3^+^ T cells (B) and CD8^+^ T cells (C) in tumors and spleens (n = 7 per group). (D) Mean fluorescence intensity (MFI) of ICAM-1 expression levels in CD8^+^ T cells in the spleens and tumors (n = 7 per group). (E) Schedule of X-ray RT in combination with FTY720 treatment in WT C57BL/6 mice bearing bilateral MC38 tumors. (F, G) Frequencies of CD4^+^ and CD8^+^ T cells among CD45^+^ cells (F), and MFI of ICAM-1 in total cells (G) of distant tumors harvested from mice after the indicated treatments as described in (E) (n = 5 per group). (H) Representative images of immunofluorescence staining of ICAM-1 in distant tumor tissues harvested from mice treated with RT or RT plus FTY720. (I, J) Representative small-animal PET images (I) and quantified tumor (distant tumor) uptake (J) of ^89^Zr-DFO-αICAM-1/Fab at 24 h postinjection in MC38 tumor-bearing mice (n = 5 per group). Distant tumors are indicated by red circles. (K, L) Individual tumor growth curves of primary tumors (K) and distant tumors (L) in WT C57BL/6 mice bearing bilateral MC38 tumors after indicated treatments as described in (E) (n = 8 per group). Data are represented as mean ± SD. *P* values were determined using unpaired Student's *t*-test (B-D, F, G, and J-L); NS, not significant (*P* > 0.05).

Although the mechanisms mediating the systemic antitumor effect of RT are poorly understood, they involve the adaptive immune system through activation of cytotoxic CD8^+^ T cells 30. Prompted by the observation that ICAM-1 is more highly expressed in tumor-infiltrating CD8^+^ T cells than in other immune cell populations 20, we hypothesized that ICAM-1 deficiency impairs the systemic antitumor effect of RT by disrupting antitumor T-cell immunity. By examining ICAM-1 levels in CD8^+^ T cells from the spleens and tumors of MC38 tumor-engrafted mice, we found that CD3^+^ or CD8^+^ T cells from tumors comprised a greater proportion of the ICAM-1^+^ population than did those from the spleens (**Figure 2A**-**C**). Furthermore, compared with the splenic CD8^+^ T cells, tumor CD8^+^ T cells exhibited markedly increased ICAM-1 expression (**Figure 2D**). Therefore, ICAM-1 expressed on tumor-infiltrating CD8^+^ T cells may facilitate their functional activation and tumor-homing ability.

To determine whether T-cell-derived ICAM-1 is essential for the systemic antitumor response to RT, we blocked T-cell infiltration into tumors via intraperitoneal (i.p.) injection of FTY720, a sphingosine analog that downregulates the sphingosine-1-phosphate receptor, blocking effector T-cell recruitment from lymphoid organs 31 (**Figure 2E**). Compared with RT alone, combined RT and FTY720 treatment significantly reduced CD4^+^ and CD8^+^ T-cell proportions among CD45^+^ cells in tumors (**Figure 2F**). Moreover, the expression of ICAM-1 in whole tumor tissues was significantly reduced upon treatment with FTY720 (*P* <0.05; **Figure 2G**,** H**).

We further examined ICAM-1 levels* in vivo* using ICAM-1-targeted PET imaging with ^89^Zr-DFO-αICAM-1/Fab, obtained by conjugating the Fab of an anti-ICAM-1 antibody (αICAM-1/Fab) with the chelator* p*-SCN-Bn-Deferoxamine (DFO) and subsequently radiolabeling with ^89^Zr (**Figure S5A**). ^89^Zr-DFO-αICAM-1/Fab can be generated with a high labeling efficiency (**Figure S5B**), and PET imaging showed that the tumor can be visualized by ^89^Zr-DFO-αICAM-1/Fab for up to 72 h postinjection (**Figure S5C**). The result of ICAM-1-targeted PET imaging revealed a significant reduction in ICAM-1 levels in the tumor following inhibition of T-cell migration with FTY720 (**Figure 2I**,** J**). Consistent with the ICAM-1 deficiency results (**Figure 1C**,** F**), FTY720 treatment abrogated the systemic antitumor effect of RT in distant tumors, without markedly impacting primary tumors (**Figure 2K**,** L**). These results demonstrate that T cells are the main source of ICAM-1 within the tumor microenvironment (TME); hence, ICAM-1 deficiency impairs the abscopal effect by disrupting antitumor T-cell immunity and ICAM-1-targeted PET imaging can reveal T cell infiltration into tumor post-RT.

### ICAM-1 impacts the proliferation and effector function of CD8^+^ T cells

**Figure 3:**
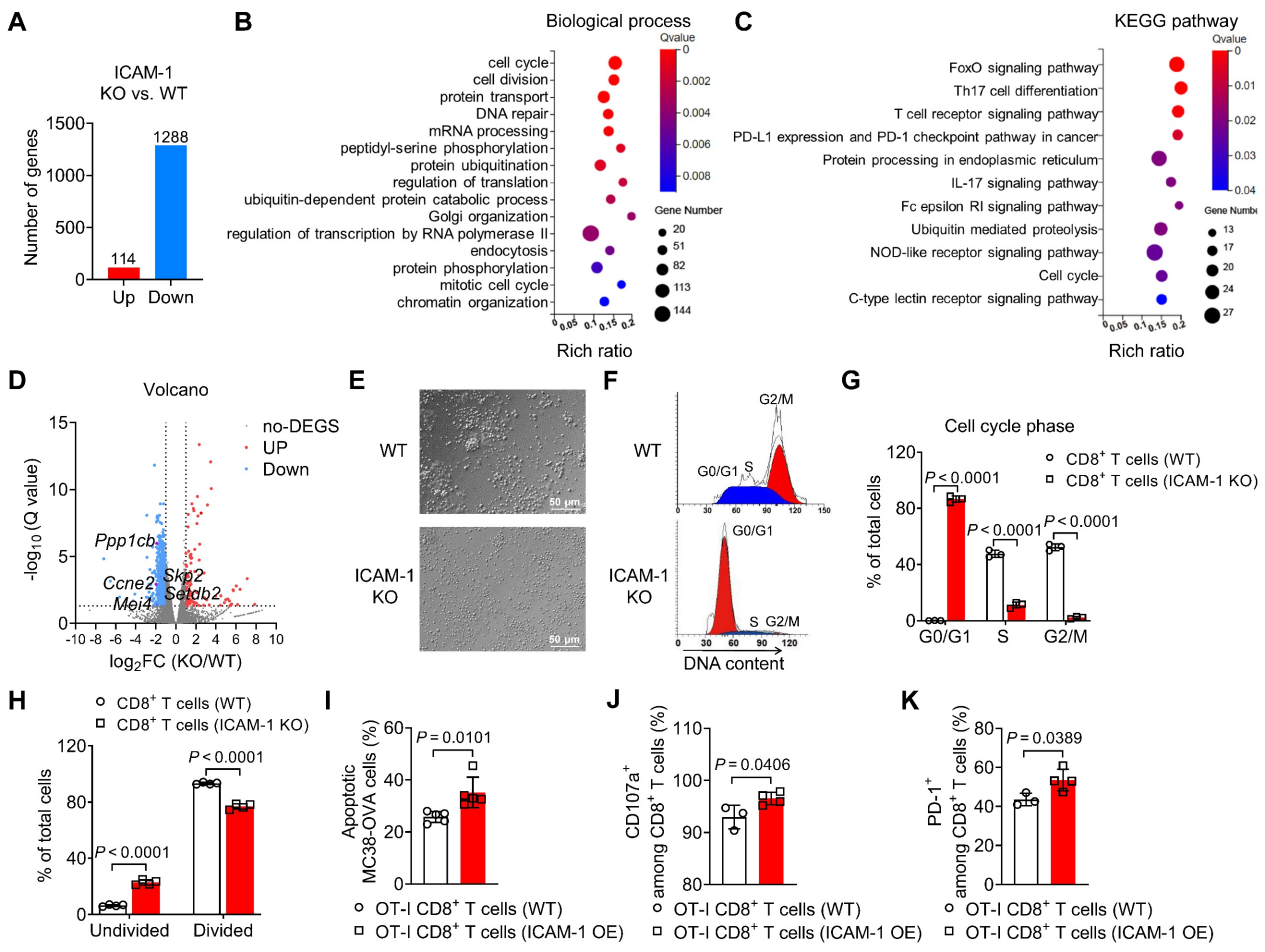
ICAM-1 regulates the cell cycle, proliferation, and cytotoxicity of CD8^+^ T cells. (A-C) RNA sequencing gene expression profiling of splenic CD8^+^ T cells isolated from WT or ICAM-1 KO mice (n = 3 per group). (A) Number of significantly upregulated or downregulated genes between WT and ICAM-1 KO CD8^+^ T cells (fold change > 2; adjusted *P* < 0.05). Significantly enriched biological process (B) and Kyoto Encyclopedia of Genes and Genomes (KEGG) pathway (C) of downregulated genes in ICAM-1 KO versus WT CD8^+^ T cells. (D) Volcano plot depicting differentially expressed genes (ICAM-1 KO versus WT CD8^+^ T cells). Genes with Q-value < 0.05 and log2 (fold change) > 1 are highlighted. Red and blue dots represent upregulated and downregulated genes, respectively, in ICAM-1 KO CD8^+^ T cells. (E) Representative microscopic images of WT or ICAM-1 KO CD8^+^ T cells. (F, G) Representative flow cytometry histograms (F) and average frequencies of each cell cycle phase (G) among WT and ICAM-1 KO CD8^+^ T cells stimulated with precoated anti-CD3 and anti-CD28 antibodies for 24 h (n = 3 per group). (H) Average frequencies of dividing and non-dividing cells among WT or ICAM-1 KO CD8^+^ T cells determined by CFSE-labeled CD8^+^ T cells stimulated with precoated anti-CD3 and anti-CD28 antibodies for 3 days (n = 4 per group). (I-K) Proportion of apoptotic MC38-OVA cells (n = 5) (I), frequencies of CD107a^+^ cells among CD8^+^ T cells (n = 3-4) (J), and frequencies of PD-1^+^ cells among CD8^+^ T cells (n = 3-4) (K) after the incubation of WT OT-I T cells or ICAM-1 OE OT-I T cells with MC38-OVA cells at an effector-to-target (E : T) ratio of 1:1 for 24 h. Data are represented as mean ± SD. *P* values were determined using unpaired Student's *t*-test (G-K).

To investigate how ICAM-1 deficiency affects gene expression in CD8^+^ T cells, we performed bulk RNA sequencing analysis of activated CD8^+^ T cells isolated from the spleens of ICAM-1 KO or WT mice. Transcriptome analysis indicated that compared with WT CD8^+^ T cells, ICAM-1 KO CD8^+^ T cells had 1288 downregulated genes and 114 upregulated genes (**Figure 3A**). Subsequently, genes downregulated in ICAM-1 KO CD8^+^ T cells were subjected to gene enrichment analyses. Gene Ontology analysis of the transcriptional differences between the ICAM-1 KO and WT control groups revealed strong enrichment in biological processes associated with the cell cycle and cell division (**Figure 3B**). Kyoto Encyclopedia of Genes and Genomes (KEGG) pathway analysis identified alterations in the FoxO signaling pathway associated with cell cycle regulation, the T-cell receptor (TCR) signaling pathway, programmed cell death-ligand 1 (PD-L1) and programmed cell death protein 1 (PD-1) checkpoint pathway in cancer (**Figure 3C**). Specifically, ICAM-1 KO CD8^+^ T cells exhibited downregulation of genes encoding cell division proteins, including *CCNE2, MEI4, PPP1CB, SETDB2*, and *SKP2* (**Figure 3D**).

Based on these observations, we investigated whether ICAM-1 deficiency affected the cell cycle and proliferation of CD8^+^ T cells. Microscopy revealed that ICAM-1 KO CD8^+^ T cells failed to form clusters during the expansion phase (**Figure 3E**), suggesting compromised cell proliferation in ICAM-1 KO CD8^+^ T cells. Flow cytometry analysis of the cell cycle phases revealed that ICAM-1 KO CD8^+^ T cells underwent cell cycle arrest in the G0/G1 phase (**Figure 3F**,** G**). Moreover, compared with WT CD8^+^ T cells, ICAM-1 KO CD8^+^ T cells showed significantly inhibited cell proliferation upon TCR stimulation (*P* <0.0001; **Figure 3H**).

We also evaluated the impact of ICAM-1 overexpression on the antitumor activity of CD8^+^ T cells. Specifically, we prepared empty vector-transduced (control) or ICAM-1 overexpressing (ICAM-1 OE) OT-I CD8^+^ T cells, cocultured them with MC38-ovalbumin (MC38-OVA) target cells and assessed their antitumor activities. Notably, ICAM-1 OE OT-I CD8^+^ T cells maintained ICAM-1 overexpression (**Figure S6A**-**C**) during coculture and exhibited increased tumor-killing capacity, degranulation, and activation (**Figure 3I**-**K**). These results suggest that ICAM-1 plays an essential role in enhancing CD8^+^ T-cell activation, proliferation, and antitumor activity.

### Genetic upregulation of ICAM-1 in CD8^+^ T cells enhances adoptive cell transfer therapy

**Figure 4:**
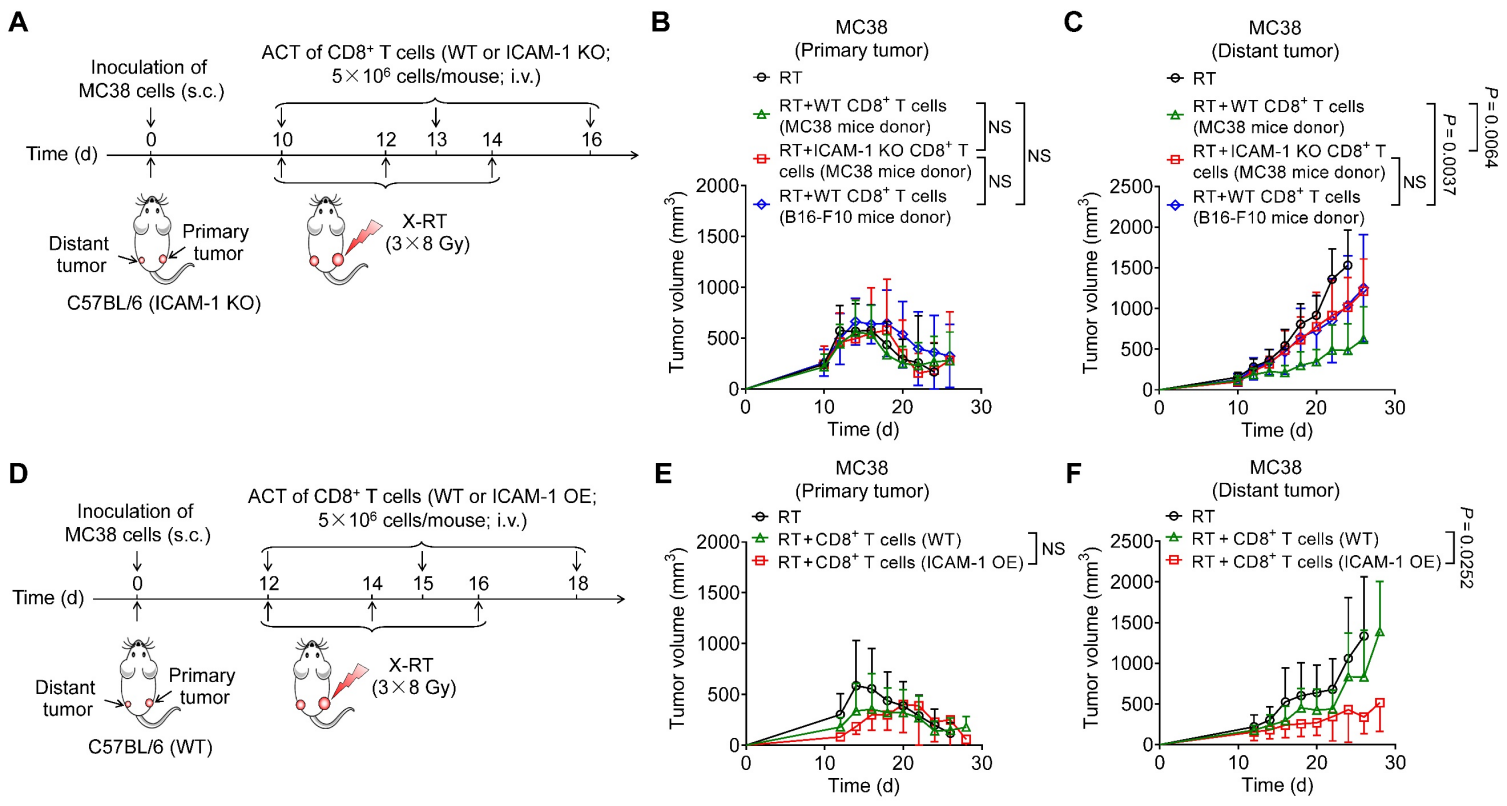
Adoptive transfer of ICAM-1^+^CD8^+^ T cells enhances the systemic antitumor effect of RT. (A) Schedule of X-ray RT combined with ACT of activated CD8^+^ T cells from MC38 tumor-bearing ICAM-1 KO C57BL/6 donor mice or activated CD8^+^ T cells from MC38 or B16-F10 tumor-bearing WT C57BL/6 donor mice in ICAM-1 KO C57BL/6 mice bearing bilateral MC38 tumors. (B, C) Average tumor growth curves of primary tumors (B) and distant tumors (C) of the MC38 tumor-bearing mice after the indicated treatments as described in (A) (n = 6 per group). (D) Schedule of RT combined with ACT of WT or ICAM-1 OE CD8^+^ T cells in WT C57BL/6 mice bearing bilateral MC38 tumors. (E, F) Average tumor growth curves of primary tumors (E) and distant tumors (F) in the MC38 tumor-bearing mice after the indicated treatments as described in (D) (n = 5-7 per group). Data are represented as mean ± SD. *P* values were determined using two-way ANOVA with Tukey's multiple-comparisons test (B, C) or unpaired Student's *t*-test (E, F); NS, not significant (*P* > 0.05).

As cross-priming of adaptive antitumor immunity by tumor-associated antigens (TAAs) is crucial for inducing the systemic antitumor effect of RT 32, we hypothesized that adoptive cell transfer (ACT) of TAA-primed CD8^+^ T cells would synergize with RT to trigger systemic antitumor reactions. To eliminate the effects of recipient endogenous ICAM-1 on CD8^+^ T cells, we isolated CD8^+^ T cells from WT donor mice bearing MC38 tumors or B16-F10 tumors and intravenously (i.v.) transplanted them into recipient ICAM-1 KO C57BL/6 mice bearing bilateral MC38 tumors, with the primary tumor receiving RT (**Figure 4A**). Our results showed that compared with recipient mice that received WT CD8^+^ T cells from mice bearing B16-F10 tumors, those that received WT CD8^+^ T cells from mice bearing MC38 tumors exhibited similar tumor inhibition in primary tumors but more potent tumor regression in distant tumors (**Figure 4B**,** C** and **Figure S7A**,** B**). We further examined how ICAM-1 expression affects the *in vivo* performance of adoptive transferred CD8^+^ T cells. RT, either alone or in combination with ACT from different sources, potently inhibited tumor growth in primary tumors (**Figure 4B** and **Figure S7A**). In contrast, RT in combination with ACT using CD8^+^ T cells from ICAM-1 KO donors, compared with those from WT donors, significantly abrogated the abscopal effect (*P* <0.001; **Figure 4C** and **Figure S7B**). RT combined with ACT did not induce body weight loss in mice, indicating a good safety profile (**Figure S7C**).

Given the critical role of ICAM-1 in CD8^+^ T cells, we hypothesized that high ICAM-1 expression in CD8^+^ T cells could augment the systemic antitumor effect of RT, and therefore conducted RT in combination with ACT in mice with more advanced (approximately 200 mm^3^) distant tumors in the bilateral MC38 tumor-bearing WT C57BL/6 mice model. The primary tumors of mice were subjected to RT and the mice were administered (i.v.) activated ICAM-1 OE CD8^+^ T cells or WT CD8^+^ T cells from naïve mice on days 12, 15, and 18 (**Figure 4D**). Adoptive administration of ICAM-1 OE CD8^+^ T cells showed comparable effects on tumor control in primary tumors to those of WT CD8^+^ T cells (**Figure 4E** and **Figure S8A**). In contrast, compared with WT CD8^+^ T cells, ICAM-1 OE CD8^+^ T cells exhibited significantly enhanced antitumor activity in distant tumors (*P* <0.05; **Figure 4F** and **Figure S8B**).

Collectively, these results indicate that ICAM-1 expression is critical for augmenting the systemic antitumor responses of CD8^+^ T cells. Moreover, the adoptive transfer of ICAM-1 OE CD8^+^ T cells could enhance the systemic antitumor effect of RT.

### Noninvasive imaging of ICAM-1 facilitates high-throughput drug screening to identify new adjuvants enhancing the systemic antitumor effect of RT

**Figure 5:**
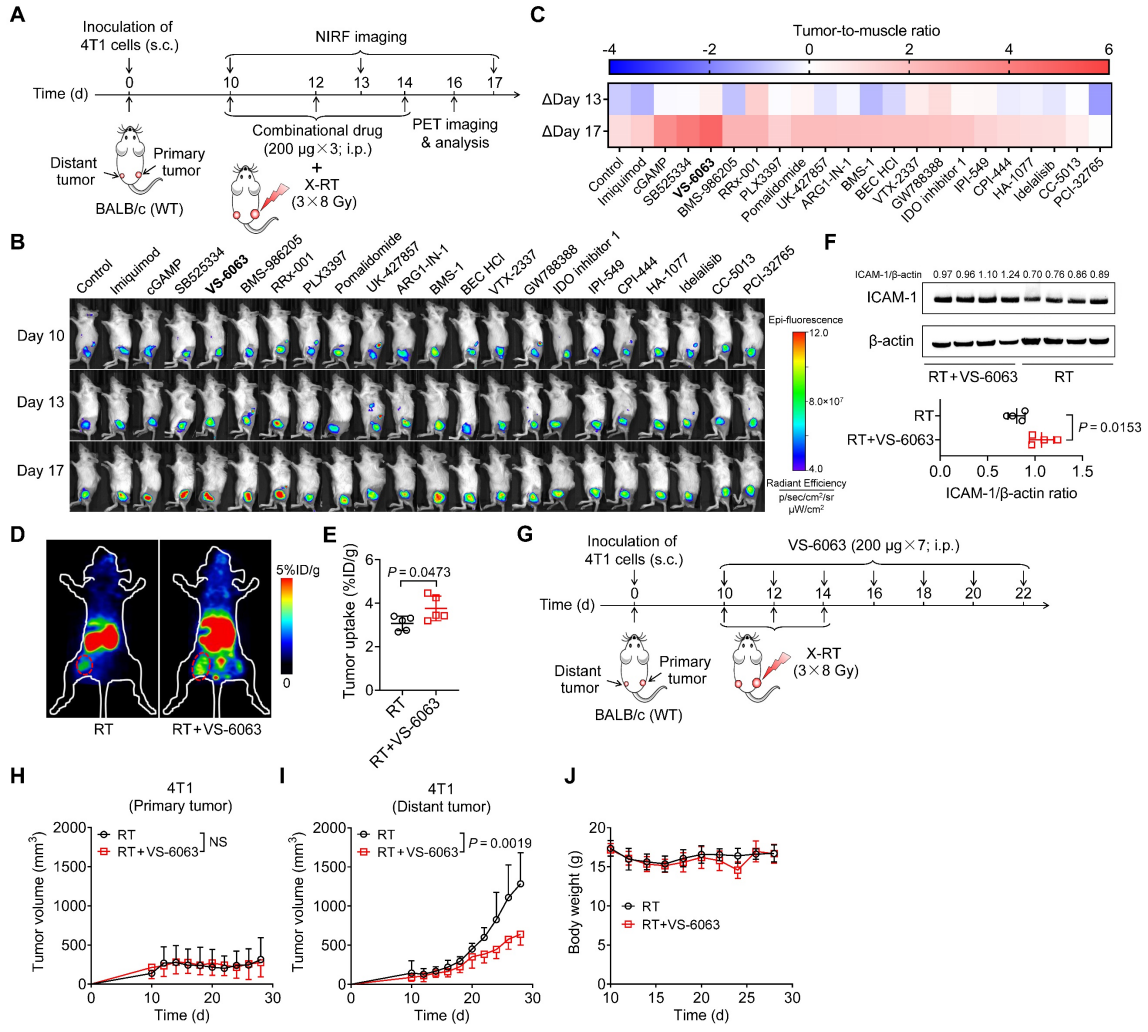
ICAM-1-targeted imaging identifies VS-6063 as a novel adjuvant to enhance the systemic antitumor effect of RT. (A) Schedule of WT BALB/c mice bearing 4T1 tumors treated with X-ray RT in combination with different small-molecule drugs, listed in Table S1. (B) Representative NIRF images of Dye-αICAM-1/Fab at 6 h postinjection in the 4T1 tumor-bearing mice on days 10, 13, and 17 after combined X-RT with different drugs as described in (A) (n = 3 per group). (C) The average Dye-αICAM-1/Fab uptake ratio of distant tumor-to-muscle at 6 h postinjection relative to the baseline values (day 10) in 4T1 tumor-bearing mice on days 13 and 17 after indicated treatments as described in (A). (D, E) Small-animal PET images (D) and quantified tumor (distant tumor) uptake (E) of ^89^Zr-DFO-αICAM-1/Fab at 24 h postinjection in 4T1 tumor-bearing mice after the indicated treatments (n = 5 per group). Distant tumors are indicated by red circles. (F) Western blotting images and quantification analysis of ICAM-1 levels in 4T1 tumor tissues on day 16 after X-RT plus VS-6063 treatment (n = 4 per group). (G) Schedule of combined RT with VS-6063 treatment in BALB/c mice bearing bilateral 4T1 tumors. (H-J) Average tumor growth curves of primary tumors (H) and distant tumors (I), and body weight (J) of 4T1 tumor-bearing mice after indicated treatments as illustrated in (G) (n = 6-7 per group). Data are represented as mean ± SD. *P* values were determined using unpaired Student's *t*-test (E, F, H, I). NS, not significant (*P* > 0.05).

Given that high expression of ICAM-1 is associated with the effectiveness of CD8^+^ T cells in mediating the antitumor immunity of RT, we next investigated whether noninvasive detection of ICAM-1 expression could be adapted to screen adjuvants capable of augmenting the systemic antitumor effect of RT, particularly in immunologically “cold” tumors. To this end, we selected several small-molecule drugs with immune activation potential that are currently undergoing clinical trials or preclinical studies (**Table S1**) and combined them with RT in a BALB/c mouse model bearing bilateral 4T1 murine breast tumors (**Figure 5A**), typically considered an immune non-responding model 18. The mice were then subjected to longitudinal ICAM-1-targeted NIRF imaging using a DyLight 800-labeled αICAM-1/Fab (Dye-αICAM-1/Fab) probe (**Figure 5B**). We then calculated the tumor (distant tumor)-to-muscle ratio of Dye-αICAM-1/Fab on days 13 and 17 relative to the baseline values on day 10. Most drugs induced an increase in the Dye-αICAM-1/Fab tumor (distant tumor)-to-muscle ratio on day 17 (**Figure 5C**). Among these drugs, the FAK inhibitor VS-6063 exhibited the most prominent capacity to upregulate ICAM-1 expression (**Figure 5B**,** C**). ICAM-1-targeted PET consistently showed that VS-6063 treatment potently upregulated ICAM-1, as evidenced by the increased uptake of ^89^Zr-DFO-αICAM-1/Fab by distant tumors (**Figure 5D**,** E**). Western blotting analysis also confirmed that VS-6063 in combination with RT, compared with RT alone, promoted the expression of ICAM-1 in distant tumors (**Figure 5F**). We further investigated the upregulation of ICAM-1 among tumor-infiltrating cell subpopulations in tumors after RT plus VS-6063 treatment. VS-6063 induced an increased proportion of ICAM-1^+^ cells among the total tumor-infiltrating cells and CD45^+^ cells without impacting CD45^-^ cells (**Figure S9A**-**C**). Consistent with prior findings 20, an increased frequency of ICAM-1^+^ cells was observed among CD8^+^ T cells but not among CD4^+^ cells (**Figure S9D**,** E**).

We then investigated whether VS-6063 could bolster systemic antitumor responses to RT in a 4T1 murine breast cancer model (**Figure 5G**). Mice treated with RT alone or in combination with VS-6063 exhibited similar primary tumor regression (**Figure 5H** and **Figure S9F**). In contrast, the administration of VS-6063 combined with RT, compared with that of RT alone, substantially inhibited tumor growth in distant tumors (**Figure 5I** and **Figure S9G**), with no body weight loss (**Figure 5J**). Taken together, these results suggest that VS-6063, screened by ICAM-1-targeted imaging, is effective in augmenting the systemic antitumor effect of RT.

### VS-6063 promotes RT by overcoming tumor stroma barriers and enhancing CD8^+^ T-cell tumor infiltration

**Figure 6:**
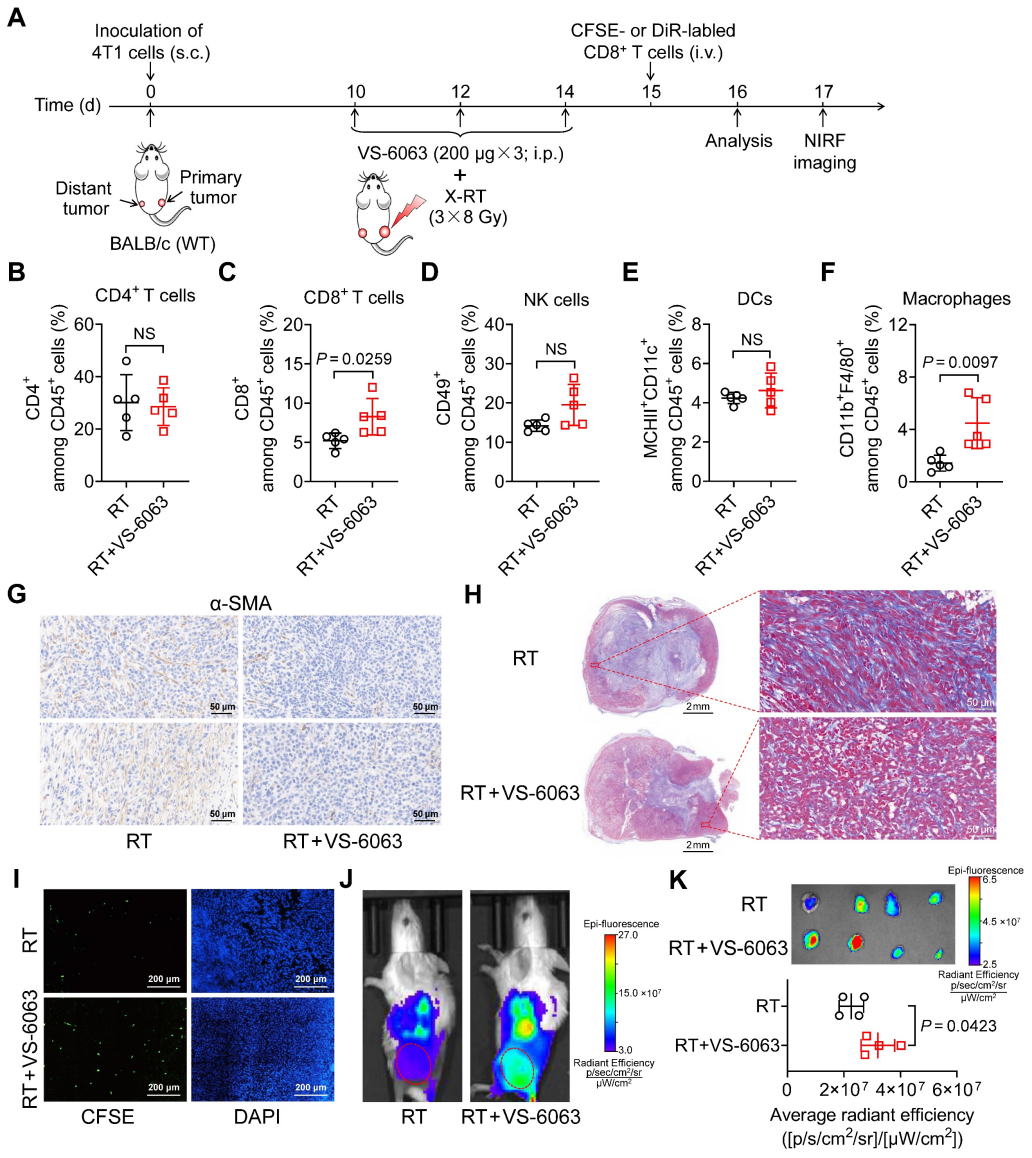
VS-6063 depletes suppressive tumor stroma to enhance CD8^+^ T-cell infiltration in tumors. (A) Schedule of combined X-ray RT with VS-6063 treatment in BALB/c mice bearing bilateral 4T1 tumors. (B-F) Frequencies of CD4^+^ cells (B), CD8^+^ cells (C), NK cells (D), DCs (E), and macrophages (F) among CD45^+^ cells in the distant tumors harvested from mice bearing 4T1 tumors after indicated treatments as illustrated in (A) (n = 5 per group). (G, H) Immunohistochemical staining of α-SMA (G) and trichrome (H) in distant tumor tissues harvested from mice bearing 4T1 tumors as illustrated in (A). (I) Representative immunofluorescence images of CFSE-labeled CD8^+^ T cells at 48 h postinjection in distant tumors after indicated treatments as illustrated in (A). (J) Representative NIRF images of DiR-labeled CD8^+^ T cells 48 h postinjection in 4T1 tumor-bearing mice after the indicated treatments as illustrated in (A). Tumors are indicated by red circles. (K)* Ex vivo* NIRF images (upper) and quantified tumor uptake (below) of DiR-labeled CD8^+^ T cells of distant tumors at 48 h postinjection in 4T1 tumor-bearing mice after indicated treatments as illustrated in (A) (n = 4 per group). Data are represented as mean ± SD. *P* values were determined using unpaired Student's *t*-test (B-F, K); NS, not significant (*P* > 0.05).

To investigate the underlying mechanisms by which VS-6063 upregulates ICAM-1 expression and enhances the systemic antitumor effect of RT in suppressing distant tumors, we compared the tumor immune infiltrates of 4T1 tumor-bearing mice treated with RT alone with those treated with a combination of RT and VS-6063 (**Figure 6A**). The addition of VS-6063 to RT increased the proportion of CD8^+^ T cells and macrophages in distant tumors while eliciting a minimal effect on the proportion of CD4^+^ T cells, NK cells, and DCs (**Figure 6B**-**F**). Recent research has indicated that CD47 blockade can activate macrophages and achieve a T-cell-independent abscopal effect in small-cell lung cancer 33. This is consistent with our results, which showed an increased frequency of macrophages in distant tumors following RT in combination with VS-6063 (**Figure 6F**). Although the frequency of macrophages increased with VS-6063 treatment, the ratio of M2-to-M1 in distant tumors remained unchanged (**Figure S10A**).

To better understand the mode of action of VS-6063, we evaluated its effects on tumor growth and tumor immune infiltration in the absence of RT (**Figure S10B**). VS-6063 alone was insufficient to control tumor progression in the 4T1 tumor model (**Figure S10C**) or significantly increase tumor immune infiltrates and the proportion of ICAM-1^+^ cells among total tumor-infiltrating cells (**Figure S10D**-**G**), ruling out VS-6063-induced cell expansion as the cause of increased infiltrates in distant tumors post-RT.

VS-6063 inhibits FAK, which is responsible for driving the formation of fibrotic desmoplastic tumor stroma composed of α-smooth muscle actin (α-SMA)^+^ cancer-associated fibroblasts (CAFs) and dense collagen-rich extracellular matrix (ECM) 34-36. As the formation of the stroma can hinder cytotoxic T-cell infiltration, we hypothesized that VS-6063 modulates CAFs and their products in tumors. Indeed, a combination of VS-6063 and RT, compared with RT alone, effectively reduced the frequency of α-SMA^+^ CAFs in distant tumors (**Figure 6G**). Moreover, VS-6063 markedly reduced collagen deposition within tumors, as indicated by trichrome staining (**Figure 6H**). These findings suggest that VS-6063 promotes suppressive stromal depletion to alleviate tumor fibrosis.

To determine whether VS-6063-induced stromal depletion promotes the infiltration of CD8^+^ cells into tumors, we administered (i.v.) carboxyfluorescein succinimidyl ester (CFSE)-labeled CD8^+^ T cells to mice bearing 4T1 tumors that received RT or RT plus VS-6063 (**Figure 6A**). Immunofluorescence staining showed that more labeled CD8^+^ T cells entered the interior of distant tumors in mice treated with RT plus VS-6063 than they did in those treated with RT alone (**Figure 6I**). We also used 1,1-dioctadecyl-3,3,3,3-tetramethylindotricarbocyanine iodide (DiR), a cell membrane fluorescent dye with a long emission wavelength, to track T cells *in vivo*. NIRF images consistently revealed that VS-6063 promoted CD8^+^ T-cell migration into the distant tumor in combination with RT (**Figure 6J**, **K**). Together, these results demonstrate that VS-6063 synergizes with RT by depleting the suppressive tumor stroma to overcome physical barriers and facilitate tumor infiltration of CD8^+^ T cells, thereby enhancing the abscopal effect.

## Discussion

In this study, we highlighted the critical role of ICAM-1 in regulating CD8^+^ T-cell activation, proliferation, infiltration, and cytotoxicity to boost the systemic antitumor effect of RT. ICAM-1 functions in multiple steps of the cancer immune cycle, and its expression on DCs collaborates with the surface receptor LFA-1 on T cells to facilitate antigen presentation 37. Meanwhile, ICAM-1 expressed in endothelial cells interacts with LFA-1 to promote the trafficking and infiltration of activated effector T cells into tumors 38,39. Our research suggests a new function of ICAM-1 in CD8^+^ T cells in the TME.

We found that tumor-infiltrating CD8^+^ T cells upregulated ICAM-1 expression to adapt to their unique function in the TME. This suggests that CD8^+^ T cells deficient in ICAM-1 fail to exert essential T-cell effector functions, including homotypic aggregation, TCR-dependent activation and proliferation, and antitumor cytotoxicity. A possible explanation for the observed effects of ICAM-1 on CD8^+^ T cells is that it provides a selective advantage by enhancing co-stimulation. In addition, ICAM-1 expression may strengthen the mechanical bond between LFA-1 and ICAM-1, thereby lowering T-cell activation thresholds in situations where TCR-peptide major histocompatibility complex (MHC) interactions are confined. Hence, future studies investigating whether ICAM-1 expression in CD8^+^ T cells modulates their co-stimulation and tunes TCR sensitivity are warranted.

We identified surface ICAM-1 on CD8^+^ T cells as a novel biomarker of T-cell activation, proliferation, tumor infiltration, and antitumor effector function. Therefore, ICAM-1-targeted PET has potential applications in contexts beyond the systemic antitumor effect of RT, such as T-cell activation and effector function imaging in immunotherapy. As a proof of concept, using ICAM-1-targeted NIRF imaging and PET, we identified the FAK inhibitor VS-6063, which significantly enhanced the antitumor immunity of RT. We showed that systemic administration of VS-6063 could efficiently deplete α-SMA^+^ CAFs and collagen-rich ECM, providing a safe and effective approach to reduce the suppressive tumor stroma without causing toxicity. As such, this drug can overcome the physical barriers of tumors and serve as a common adjuvant capable of synergistically improving the limited boost of the RT systemic antitumor effect and promoting tumor homing of therapeutic T cells in ACT, including chimeric antigen receptor T-cell (CAR-T) therapy. This combination enhances the systemic antitumor effect of RT, confirming the predictive value of ICAM-1-targeted imaging. Given that PET radiotracers are highly clinically translatable, further optimization of ICAM-1-based PET radiotracers is warranted for an effective noninvasive strategy to guide combinational RT in patients with cancer to facilitate precision therapy.

## Conclusion

In this study, we found that ICAM-1 deficiency interfered with the ability of RT to induce the abscopal effect by disrupting the antitumor immunity of CD8^+^ T cells, impairing their proliferative capacity and cytotoxic function. Our findings provide mechanistic insights into how ICAM-1 augments the proliferation and effector function of CD8^+^ T cells and influences T cell-based antitumor immunity. Moreover, we proposed a noninvasive imaging strategy using ICAM-1-targeted NIRF imaging or clinically relevant PET to guide drug screening, select rational drugs for combination therapy with RT, and boost robust antitumor immunity. Collectively, the findings of this study indicate that monitoring tumor infiltration by ICAM-1^+^CD8^+^ T cells using ICAM-1-targeted noninvasive imaging provides a novel means to develop effective RT combination therapies and increases the frequency of the abscopal effect in clinical settings.

## Materials and Methods

### Mice

Five- to six-week-old female C57BL/6 mice and BALB/c mice were purchased from the Department of Laboratory Animal Science at Peking University (Beijing, China). TCR-transgenic OT-I mice (C57BL/6-Tg (TcraTcrb)1100Mjb/J) and ICAM-1 KO mice in C57BL/6 background were obtained from Shanghai Model Organisms Center, Inc. (Shanghai, China). The experimental procedures in the mouse studies were conducted in accordance with the protocols approved by the Institutional Animal Care and Use Committee at Peking University.

### Cell culture and animal models

MC38 and MC38-OVA murine colon cancer cells, B16-F10 murine melanoma cells, and 4T1 murine breast cancer cells were purchased from the American Type Culture Collection. MC38, MC38-OVA, and B16-F10 cells were cultured in Dulbecco modified Eagle medium (DMEM; Invitrogen, Carlsbad, CA). 4T1 cells were cultured in RPMI-1640 medium (Invitrogen). All cells were grown in a medium supplemented with fetal bovine serum (10% v/v) at 37°C in a humidified atmosphere containing 5% CO_2_.

To establish the 4T1 tumor-bearing model, 1 × 10^6^ tumor cells were injected subcutaneously into the flanks of BALB/c mice. To establish MC38 and B16-F10 tumor -bearing models, 1×10^6^ tumor cells were subcutaneously injected into the flanks of WT or ICAM-1-KO mice C57BL/6 mice. For the bilateral tumor-bearing model, 1 × 10^6^ tumor cells were subcutaneously injected into the right hind flank (primary tumor) and 5 × 10^5^ cells in the left hind flank (distant tumor) of mice. Tumor growth was measured using a caliper every other day, and tumor size was calculated using the following formula: volume = length × width^2^/2.

### Preparation of T cells

The spleens from donor mice, including naïve WT mice, MC38 or B16-F10 tumor-bearing WT or ICAM-1 KO C57BL/6 mice, and OT-I mice, were disintegrated and filtered through a strainer. Red blood cells were lysed with erythrocyte lysis buffer (BioLegend, San Diego, CA), and the splenocytes were washed and resuspended at a cell density of 2 × 10^6^ cells/mL in complete RPMI-1640 medium supplemented with interleukin-2 (IL-2; 10 ng/mL, Peprotech, Rocky Hill, NJ). For WT T cells, activation was performed in plates precoated with anti-CD3 (1 µg/mL; clone 145-2C11, BioLegend) and anti-CD28 (2 µg/mL; clone 37.51, BioLegend) antibodies. On day 3, the T cells were enriched with mouse CD8 MicroBeads and MACS LS columns (Cat#: 130-116-478, Miltenyi Biotec; Bergisch Gladbach, Germany). For ICAM-1 OE CD8^+^ T cells, WT CD8^+^ T cells were transduced by lentiviral transfection as per the manufacturer's instructions (SyngenTech, Beijing, China). The percentage of transduced cells was assessed by flow cytometry to detect green fluorescent protein expression.

For ACT or *in vitro* cell proliferation studies, activated WT (5 × 10^6^), ICAM-1 KO (5 × 10^6^), or ICAM-1 OE CD8^+^ T cells (5 × 10^6^) were used. OT-I T cells were resuspended (2 × 10^6^ cells/mL) in a complete RPMI-1640 medium supplemented with mouse IL-2 (10 ng/mL) and OVA_257-264_ peptide (1 μM, Invitrogen). ICAM-1 OE OT-I T cells were prepared similarly to that for ICAM-1 OE WT T cells. Peptide-primed OT-I T cells were used for the coculture assay.

### Radiotherapy

Therapeutic irradiation was performed using an X-ray irradiator (dose rate of 4.12 Gy/min; RS2000 PRO; 160 kV, 25 mA; Rad Source Technologies). The mice were anesthetized by i.p. injection of tribromoethanol (Bidepharm, Shanghai, China). Irradiation was delivered to target tumors with 5 mm margins at a total dose of 24 Gy in three fractions every other day while sparing the adjacent normal tissue.

### Antitumor therapy studies

Mice bearing bilateral MC38, B16-F10, or 4T1 tumors were subjected to RT in combination with other antitumor therapies. For MC38 or B16-F10 bilateral tumor-bearing ICAM-1 KO or WT C57BL/6 mice or 4T1 bilateral tumor-bearing BALB/c mice, the right hind flank tumors (primary tumors) were irradiated with a total dose of 24 Gy (three doses of 8 Gy) or non-irradiated.

For the anti-ICAM-1 blocking experiment, 50 μg of anti-ICAM-1 antibody (clone YN1/1.7.4, BioXcell, West Lebanon, NH) or an equal amount of IgG isotype-matched control was intratumorally injected into the left hind flank tumors on days 8, 10, 12, and 14. For the fingolimod (FTY720) treatment, MC38 tumor-bearing mice were injected (i.p.) with 40 μg of FTY720 on day 9 and 20 μg of FTY720 daily until day 28. For ACT, MC38 tumor-bearing ICAM-1 KO mice were administered (i.v.) activated CD8^+^ T cells from MC38 tumor-bearing ICAM-1 KO C57BL/6 donor mice (5 × 10^6^) or activated CD8^+^ T cells from MC38 or B16-F10 tumor-bearing C57BL/6 WT donor mice (5 × 10^6^) on days 10, 13, and 16. MC38 tumor-bearing WT mice were administered (i.v.) activated WT or ICAM-1 OE CD8^+^ T cells (5 × 10^6^) on days 12, 15, and 18. For small-molecule drug treatment, 4T1 tumor-bearing mice were injected (i.p.) with 200 µg of VS-6063 (Selleckchem, Houston, TX) every other day from days 10 to 22.

### Analyses of immune cells in tumors and spleens

C57BL/6 mice bearing either single or bilateral MC38 tumors received three fractions of 8 Gy radiation to the right hind flank tumors or were non-irradiated. From days 8 to 14, mice were intratumorally administered PBS, anti-ICAM-1 antibody (50 µg), or IgG isotype-matched control (50 µg) every other day. On day 17, the mice were euthanized, and the tumors and spleens were collected.

In a separate study, BALB/c mice with either single or bilateral 4T1 tumors received three fractions of 8 Gy radiation to the right hind flank tumors or were non-irradiated. From days 10 to 14, they received i.p. injections of PBS control or 200 μg of VS-6063 (Selleckchem) every other day. On day 16, the mice were euthanized, and the tumors were harvested.

Tumor samples were mechanically minced and digested in RPMI-1640 medium (Invitrogen) supplemented with 10 U/mL collagenase I, 400 U/mL collagenase IV, and 30 U/mL DNase (Yuanye Bio-Technology, Shanghai, China) at 37°C for 1 h. The spleens were dissected and filtered through a strainer. Red blood cells in the digested tumor samples and spleens were lysed using erythrocyte lysis buffer (BioLegend). Tumor-infiltrating cells and spleen cells were resuspended in PBS containing 0.5% w/v bovine serum albumin (BSA). The collected cells were then stained and analyzed using flow cytometry.

### Flow cytometric analyses

The fluorescently labeled antibodies used for flow cytometric analysis are list in **Table S2**. For surface marker staining, cells were blocked with BSA and incubated with the indicated antibodies at 4°C for 20 min. The cells were washed and resuspended in PBS containing BSA (0.2%, w/v) for flow cytometric analysis. For cell cycle staining, cells were stained with propidium iodide using a cell cycle and apoptosis analysis kit (Beyotime, Shanghai, China). Cell detection was performed using an LSRFortessa flow cytometer (Becton Dickinson, Sunnyvale, CA), and analyses were performed using FlowJo 10.6.1 (Tree Star).

### *In vitro* T-cell proliferation assay

CD8^+^ T cells were isolated from the spleens of ICAM-1 WT or ICAM-1 KO C57BL/6 mice using mouse CD8 Microbeads and MACS LS columns (Miltenyi Biotec) according to the manufacturer's instructions. Isolated T cells were suspended in PBS containing 5 μM CFSE (Thermo Fisher Scientific, Waltham, MA) and incubated at 37°C for 10 min. After washing, cells were resuspended in RPMI-1640 medium containing IL-2 (10 ng/mL). These CFSE-labeled T cells were then stimulated in plates precoated with anti-CD3 (1 µg/mL; clone 145-2C11, BioLegend) and anti-CD28 (2 µg/mL; clone 37.51, BioLegend) antibodies. Following a 3-day coculture, the cells were collected for flow cytometric analysis, and T-cell division was determined via CFSE dilution.

### *In vitro* coculture of OT-I T cells with MC38-OVA cells

MC38-OVA tumor cells were seeded at a density of 8 × 10^4^ cells/well in 12-well plates containing complete DMEM (Invitrogen) and incubated overnight. The tumor cell culture medium was aspirated, and either ICAM-1 overexpressing OT-I CD8^+^ T cells (ICAM-1 OE) or control OT-I CD8^+^ T cells suspended in RPMI-1640 medium supplemented with IL-2 (10 ng/mL) were added to the tumor cells at an effector-to-target ratio of 1:1. After a further 24 h coculture, the cells were collected, and tumor cell apoptosis was determined using an annexin V-FITC apoptosis detection kit (Solarbio, Beijing, China); T-cell phenotype was analyzed using flow cytometry.

### Immunofluorescence staining

Frozen MC38 or 4T1 tumor sections were fixed with ice-cold acetone and blocked in 5% BSA (in PBS). Subsequently, the tumor sections were incubated with anti-ICAM-1 primary antibodies (clone YN1/1.7.4; 1:100, Santa Cruz Biotechnology, Santa Cruz, CA) overnight at 4 °C and incubated with DyLight549 conjugated anti-rat IgG secondary antibody (clone E032340; 1:200; EarthOx, Millbrae, CA) followed by 4',6-diamidino-2-phenylindole (1:1000; Biotium, Fremont, CA). After washing, the tumor sections were visualized under a confocal microscope (Leica, Wetzlar, Germany).

### Immunohistochemistry

The 4T1 tumor tissues were fixed in 10% neutral formalin, embedded in paraffin, and sectioned at 5-µm thickness. For tumor α-SMA staining, tumor sections were deparaffinized, endogenous peroxidase activity was abolished, and antigens were retrieved. After incubating with an anti-mouse α-SMA antibody (1:200; Abcam, Cambridge, MA) overnight at 4°C, the tumor sections were incubated with horseradish peroxidase (HRP)-conjugated secondary antibody and visualized by incubation with a diaminobenzidine substrate.

For Masson's trichrome staining, the tumor tissue sections were stained with Masson's trichrome (muscle fibers are stained red and collagen fibers, blue or green), according to the manufacturer's instructions. Images were acquired using a DM5000B microscope (Leica).

### Western blotting

Proteins from tumor tissues were extracted using radioimmunoprecipitation assay buffer and protein concentrations were determined using a BCA assay kit (Thermo Fisher Scientific). The proteins were separated by sodium dodecyl sulfate-polyacrylamide gel electrophoresis, transferred to a polyvinylidene difluoride membrane (Invitrogen), and blocked in 5% skim milk for 1 h. The membrane was incubated overnight at 4°C with an anti-ICAM-1 primary antibody (1:500; clone YN1/1.7.4, Santa Cruz), followed by incubation with HRP-conjugated anti-rat IgG secondary antibody (Beyotime Biotechnology, 1:10000) for 1 h. Images were captured using a Molecular Imager PharosFX™ Plus System. (Bio-Rad Laboratories, Hercules, CA).

### Preparation of Dye-αICAM-1/Fab and ^89^Zr-DFO-αICAM-1/Fab

ICAM-1-specific NIRF and PET imaging probes were synthesized as previously described 20,40. Briefly, the anti-ICAM-1 antibody (clone YN1/1.7.4, BioXcell) was digested using a Fab preparation kit (Thermo Fisher Scientific), and the Fab fragment of αICAM-1 (αICAM-1/Fab) was obtained. To synthesize the NIRF imaging probe, αICAM-1/Fab was conjugated with the DyLight 800-NHS ester (Thermo Fisher Scientific) in the NaHCO_3_ buffer (pH 8.4) to yield the Dye-αICAM-1/Fab probe.

To synthesize ^89^Zr-labeled αICAM-1/Fab, αICAM-1/Fab in PBS was adjusted to pH 8.5-9.0 using 0.1 M Na_2_CO_3_. The pH-adjusted αICAM-1/Fab was conjugated with the bifunctional chelator DFO (Macrocyclics, Dallas, TX) at a molar ratio of 1:10, followed by pH readjustment to 8.5-9.0. DFO-αICAM-1/Fab was purified using a PD-10 desalting column (GE Healthcare, Piscataway, NJ). For radiolabeling, 74 MBq of ^89^Zr-oxalate was buffered with 0.5 M HEPES (pH = 7.0, Biorigin, Beijing, China), and 50 μg of DFO-αICAM-1/Fab was added to the radioactive solution. After shaking for 1 h at 37°C, ^89^Zr-DFO-αICAM-1/Fab was purified using a desalting column, and its purity was confirmed using instant thin-layer chromatography.

### *In vivo* small-animal NIRF and PET imaging

To perform *in vivo* NIRF imaging, mice were injected (i.v.) with Dye-αICAM-1/Fab (0.5 nmol per mouse) and imaged 6 h postinjection using the IVIS Spectrum In Vivo Imaging System (Xenogen, Alameda, CA). Excitation and emission wavelengths were 745 and 800 nm, respectively. The region of interest (ROI) for each tumor was delineated using the LivingImage software (Xenogen).

For *in vivo* small-animal PET imaging, ^89^Zr-DFO-αICAM-1/Fab (3.7 MBq/~5 μg per mouse) was administered (i.v.) to MC38 or 4T1 tumor-bearing mice. At 6, 12, 24, 48, and 72 h postinjection, 10-minute static PET scans were acquired using a Super Nova PET/CT scanner (Pingseng Healthcare, Shanghai, China). PET images were analyzed and the ROI-derived percentage of the injected dose per gram of tissue (%ID/g) was calculated as previously described 18.

### RNA sequencing analysis

CD8^+^ T cells were sorted from the spleens of WT or ICAM-1 KO C57BL/6 mice using mouse CD8 Microbeads and MACS LS columns (Miltenyi Biotec), according to the manufacturer's instructions. RNA was extracted using the TRIzol reagent (Invitrogen) according to the manufacturer's protocol (*n* = 3). RNA samples were processed for library preparation and sequenced using the BGISEQ-500 system at the Beijing Genomics Institute. Raw data were subjected to standard RNA sequencing analysis pipeline processing. HISAT2 and Bowtie2 were used to align the clean tag reads to the reference genome and reference genes. Matched reads were calculated and normalized using the RSEM software. Differential expression analysis of raw counts was conducted using the DESeq2 R library. Genes identified as significantly differentially expressed were characterized and analyzed at https://biosys.bgi.com. The adjusted *P-*value cut-offs for the lists of genes were as follows: ICAM-1 KO versus WT: 1402 differentially expressed genes with an adjusted *P-*value < 0.05 and fold change > 2. Volcano plots were created using the log2 fold change of genes determined as differentially expressed by DESeq2 (adjusted* P*-value < 0.05).

### ICAM-1-targeted imaging for drug screening

To evaluate the capacity of small-molecule drugs to upregulate ICAM-1 when combined with RT, we tested several drugs, as listed in **Table S1**. Imiquimod (IMQ, Sigma-Aldrich, St. Louis, MO) was used as a positive control 20. Mice were injected (i.p.) with 200 µg of small-molecule drugs in a 100 µL compound mixture (5% DMSO + 40% polyethylene glycol 300 + 5% Tween 80 + 50% saline) on days 10, 12, and 14. On days 10, 13, and 17, the mice were injected (i.v.) with Dye-αICAM-1/Fab (0.5 nmol per mouse) and subjected to NIRF imaging.

### T-cell tracking assay

To track T-cell migration, sorted CD8^+^ T cells were labeled with CFSE (Thermo Fisher Scientific) or DiR (AAT Bioquest, Sunnyvale, CA). On day 15, 4T1 tumor-bearing mice were injected via tail vein with CFSE-labeled CD8^+^ T cells or DiR-labeled T cells (5 × 10^6^). On day 17, the mice that received CFSE-labeled CD8^+^ T cells were euthanized, and the tumors were harvested and cut into sections for immunofluorescence staining. The mice that received DiR-labeled T cells were subjected to NIRF imaging (excitation wavelength: 745 nm, emission wavelength: 800 nm). Immediately after *in vivo* imaging, the mice were euthanized, tumors were harvested, and* ex vivo* NIRF imaging was performed.

### Statistical analysis

Statistical analysis was performed using GraphPad Prism 9 (GraphPad Software, San Diego, CA). Quantitative data are presented as mean ± standard deviation (SD). Comparisons of two groups were performed using two-tailed unpaired Student's *t*-test. Tumor growth curves over time were compared using two-way analysis of variance (ANOVA) with Tukey's multiple-comparisons test. The statistical method for the data is also specified in the corresponding figure legends. *P-*values of < 0.05 were considered statistically significant.

## Supplementary Material

Supplementary figures and tables.
