# Supplementary material for: Annotation of CD8+ T-cell function via ICAM-1 imaging identifies FAK inhibition as an adjuvant to augment the antitumor immunity of radiotherapy
Source: Theranostics. 2024 Jan 1;14(2):699–713. doi: 10.7150/thno.90709 (PMC10758046; doi:10.7150/thno.90709)
Supplement: Supplementary file 1 — Supplementary figures and tables. [file thnov14p0699s1.pdf]

Supplementary Materials for

Annotation of CD8$^+$ T-cell function via ICAM-1 imaging identifies FAK inhibition as an adjuvant to augment the antitumor immunity of radiotherapy

Ting Zhang[1#], Yining Zhang[1#], Yang Zhao[1], Rui Song[1], Yanpu Wang[1], Kui Li[1], Haoyi Zhou[1], Feng Wang[2], Shixin Zhou[3], Meixin Zhao[4], Hua Zhu[2], Weifang Zhang[4]*, Zhi Yang[2]*, Zhaofei Liu[1,2,4,5,6]*

[1]Department of Radiation Medicine, School of Basic Medical Sciences, Peking University Health Science Center, Beijing 100191, China

[2]Key Laboratory of Carcinogenesis and Translational Research (Ministry of Education/Beijing), Key Laboratory for Research and Evaluation of Radiopharmaceuticals (National Medical Products Administration), Department of Nuclear Medicine, Peking University Cancer Hospital and Institute, Beijing 100142, China

[3]Department of Cell Biology, School of Basic Medical Sciences, Peking University Health Science Center, Beijing 100191, China

[4]Department of Nuclear Medicine, Peking University Third Hospital, Beijing 100191, China

[5]State Key Laboratory of Vascular Homeostasis and Remodeling, Peking University, Beijing 100191, China

[6]Peking University-Yunnan Baiyao International Medical Research Center, Beijing 100191, China.

[#]These authors contributed equally to this work.

*Corresponding authors: Zhaofei Liu, E-mail: liuzf@bjmu.edu.cn; Zhi Yang, Email: pekyz@163.com; Weifang Zhang, Email: tsy1997@126.com

This PDF file includes:

Figure S1-S10

Table S1-S2

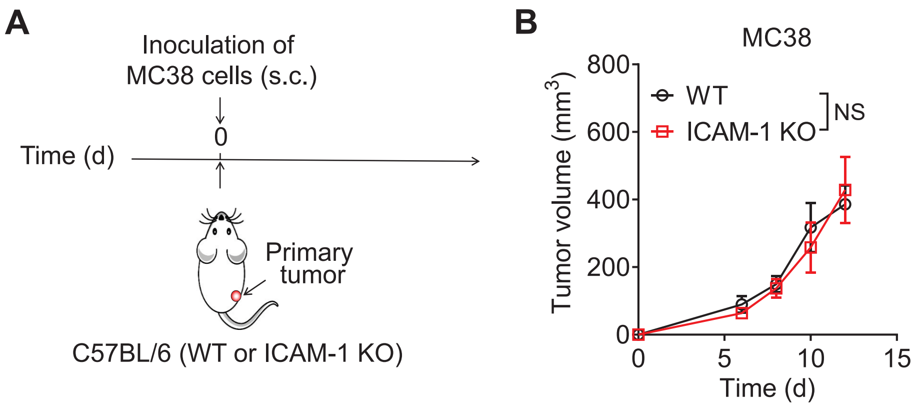

Figure S1. ICAM-1 KO has negligible effects on tumor progression. (A, B) Schematic illustration of the establishment of MC38 tumor model (A) and average tumor growth curves (B) in WT or ICAM-1 KO C57BL/6 mice (n = 5–6 per group). Data are represented as mean ± SD. *P* values were determined using unpaired Student's *t*-test (B); NS, not significant ($P > 0.05$).

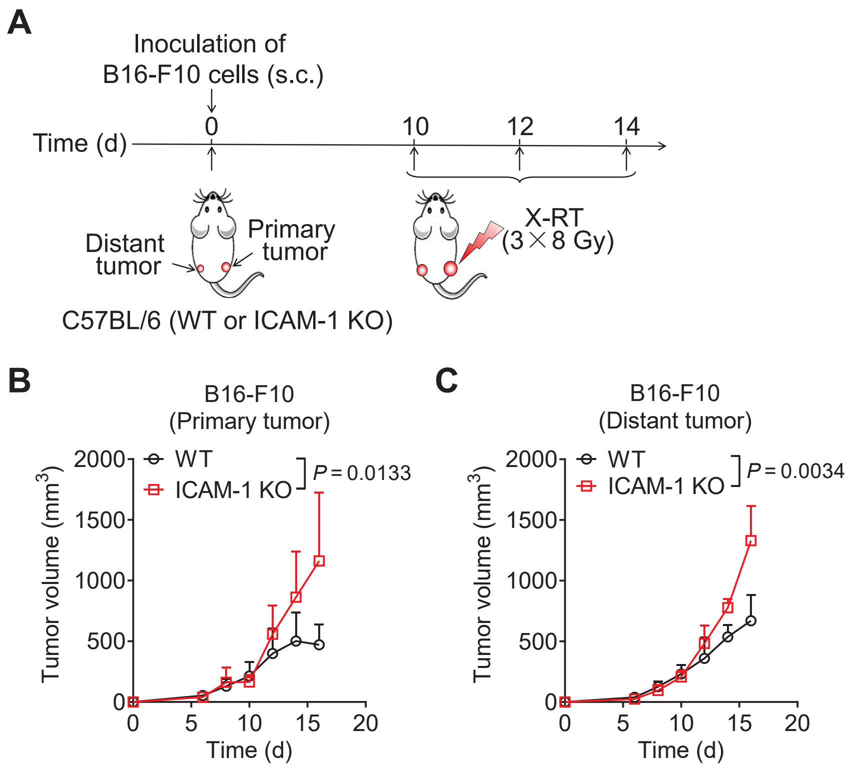

Figure S2. ICAM-1 deficiency impairs the abscopal effects of RT in the B16-F10 tumor model. (A) Schedule of X-ray RT in the WT or ICAM-1 KO C57BL/6 mice bearing bilateral B16-F10 tumors. (B, C) Average tumor growth curves of the primary (B) and distant tumors (C) of mice bearing B16-F10 tumors after RT as illustrated in (A) (n = 3–7 per group). Data are represented as mean ± SD. *P* values were determined using unpaired Student's *t*-test (B, C).

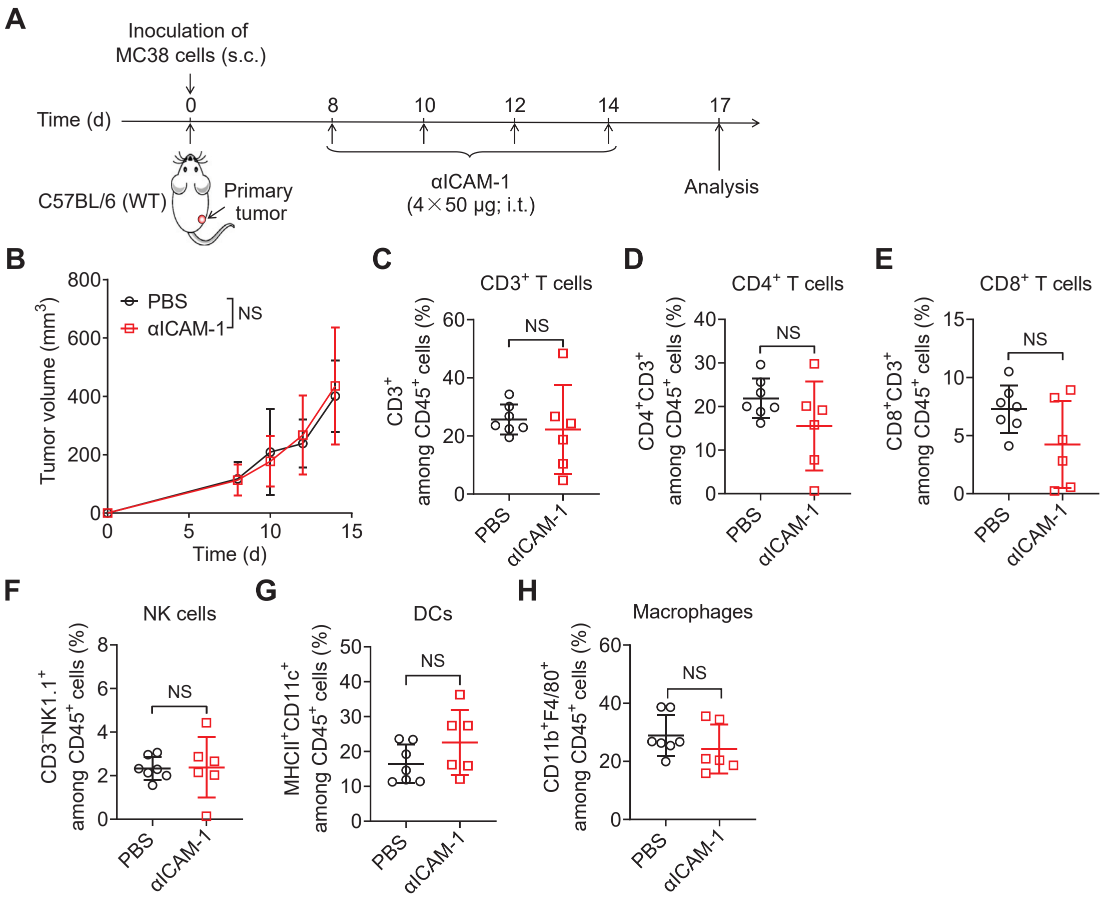

Figure S3. ICAM-1 blockade alone has negligible effects on tumor progression and immune infiltration of tumors. (A) Schematic illustration of ICAM-1 blockade with an anti-ICAM-1 antibody in WT C57BL/6 mice bearing MC38 tumors. (B) Average tumor growth curves of MC38 tumor-bearing mice after the indicated treatments (n = 8 per group). (C–H) Frequencies of $CD3^+$ T cells (C), $CD4^+$ T cells (D), $CD8^+$ T cells (E), NK cells (F), DCs (G), and macrophages (H) among $CD45^+$ cells in tumor tissues harvested from MC38 tumor-bearing mice after the indicated treatments as illustrated in (A) (n = 6–7 per group). Data are represented as mean ± SD. *P* values were determined using unpaired Student's *t*-test (B–H); NS, not significant ($P > 0.05$).

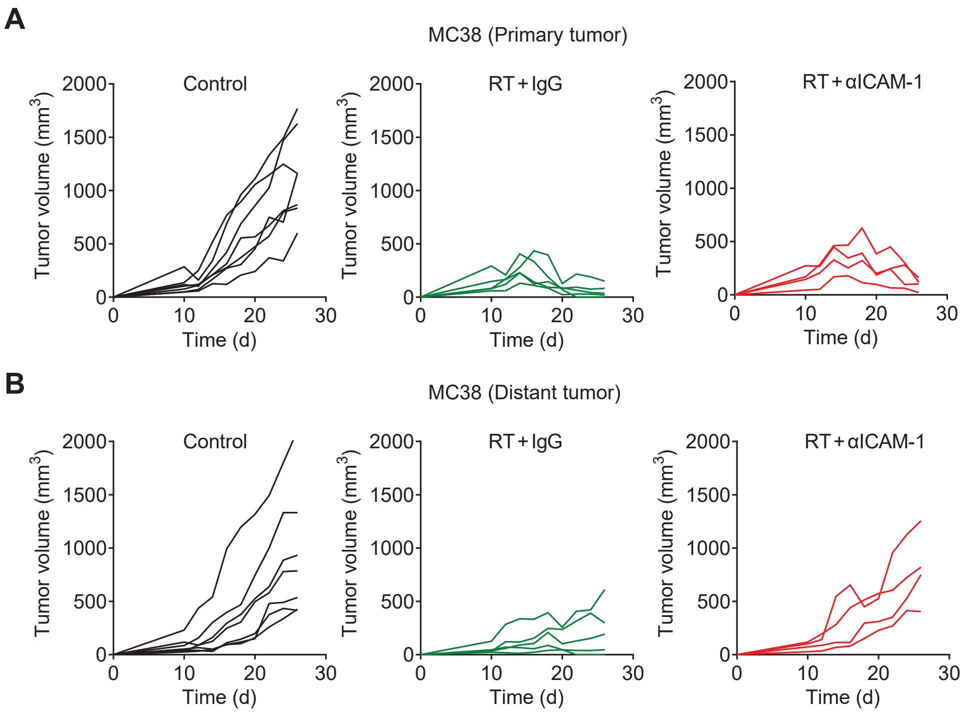

Figure S4. ICAM-1 blockade impairs the abscopal effects of RT in the MC38 tumor model. (A, B) Individual tumor growth curves of primary tumors (A) and distant tumors (B) in mice bearing the MC38 tumors after indicated treatments as illustrated in Figure 1D in the main text (n = 4–7 per group). Data related to Figure 1E–F in the main text.

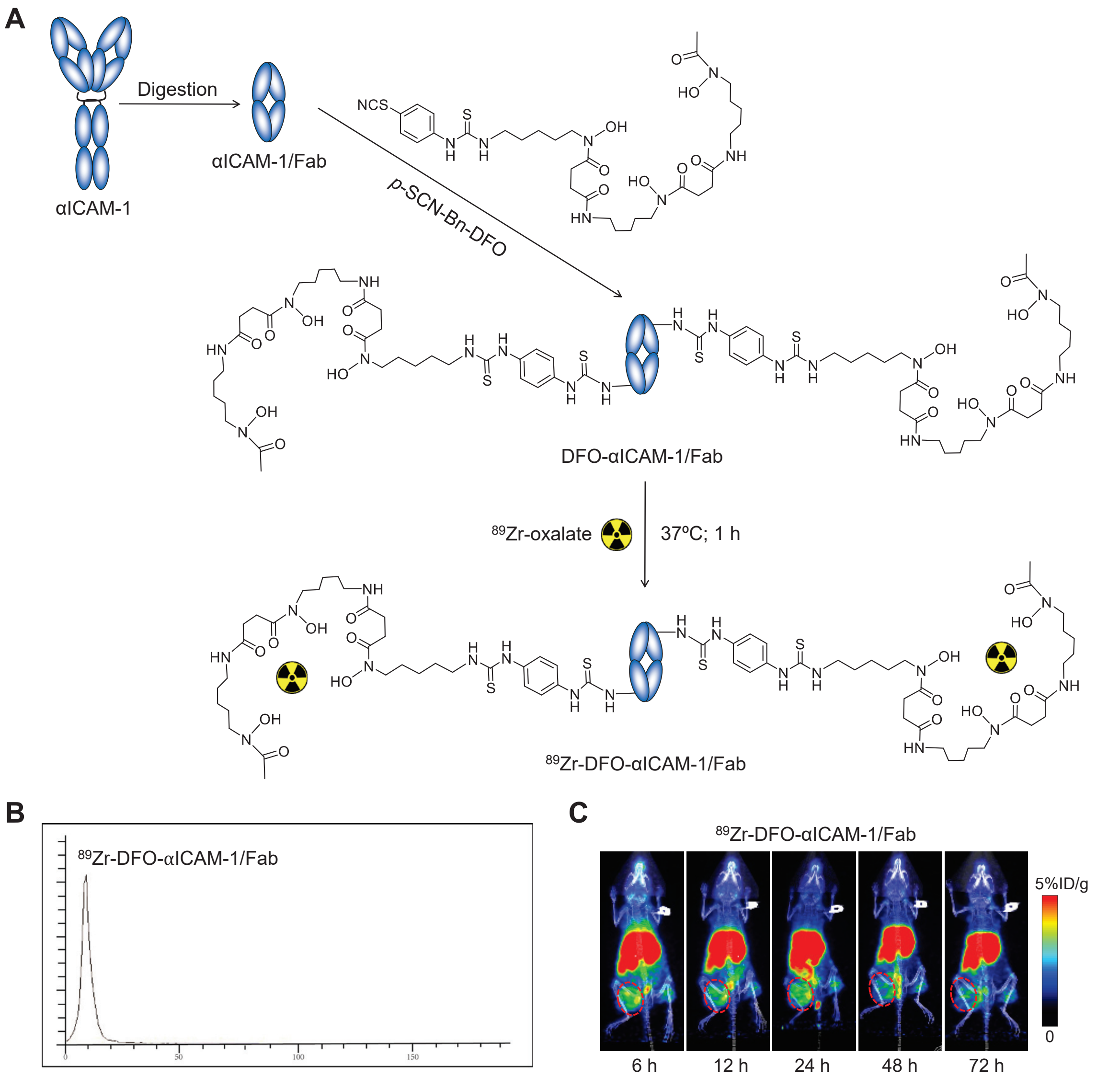

Figure S5. Synthesis and characterization of ^{89}Zr-DFO-αICAM-1/Fab. (A) Preparation of the anti-ICAM-1 antibody Fab fragment (αICAM-1/Fab), desferrioxamine (DFO) conjugation, and ^{89}Zr radiolabeling to generate ^{89}Zr-DFO-αICAM-1/Fab. (B) The labeling efficiency of ^{89}Zr-DFO-αICAM-1/Fab as determined using ITLC. (C) Small-animal PET/CT images of ^{89}Zr-DFO-αICAM-1/Fab at 6, 12, 24, 48, and 72 h postinjection in 4T1 tumor-bearing mice. Tumors are indicated by red circles.

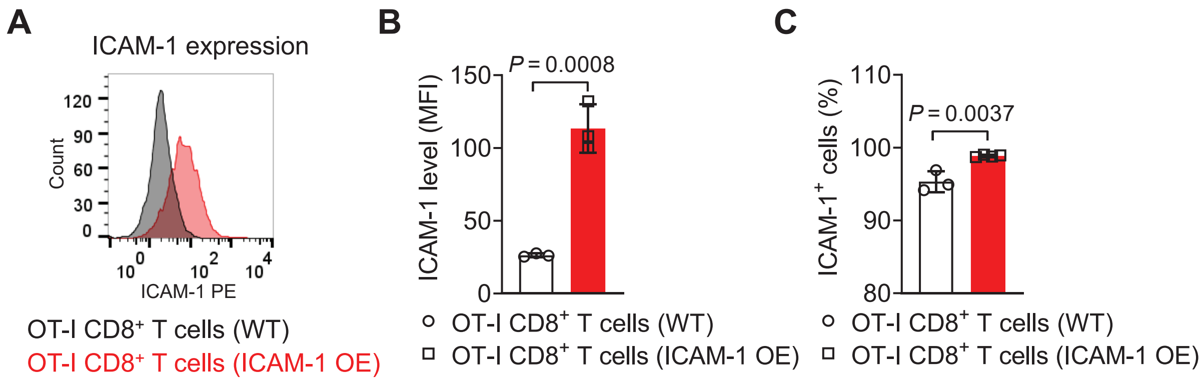

Figure S6. Characterization of ICAM-1 OE OT-I $CD8^+$ T cells. (A, B) Representative flow cytometry histograms (A) and mean fluorescence intensity (MFI) (B) of ICAM-1 expression levels on WT or ICAM-1 OE OT-I $CD8^+$ T cells (n = 3 per group). (C) Frequencies of ICAM-1^+ cells among WT or ICAM-1 OE OT-I $CD8^+$ T cells (n = 3–4 per group). Data are represented as mean ± SD. *P* values were determined using unpaired Student's *t*-test (B, C).

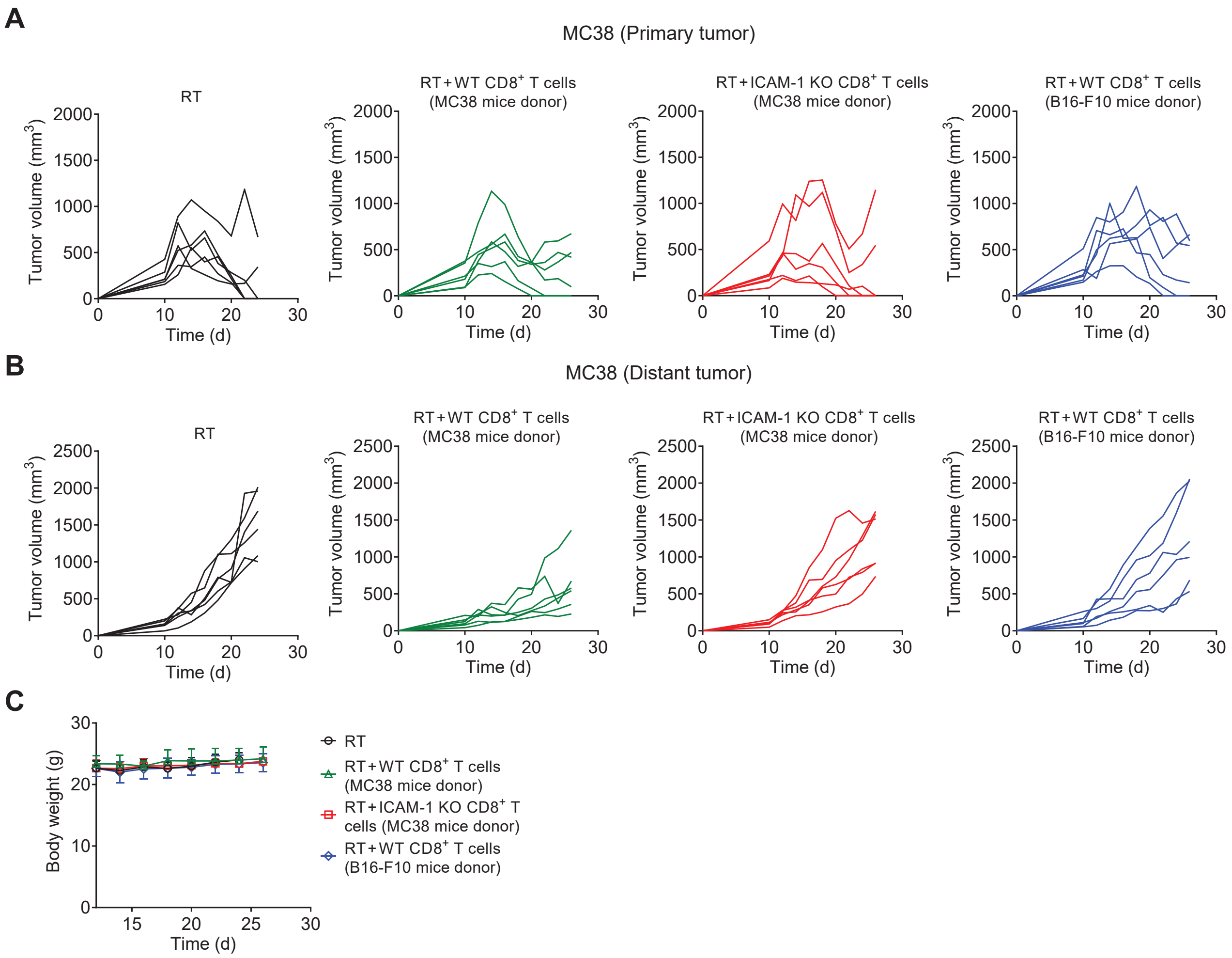

Figure S7. ICAM-1 KO impairs the abscopal effect of RT in combination with ACT therapy of CD8^{+} T cells. (A–C) Individual tumor growth curves of primary tumors (A), distant tumors (B), and body weight (C) of the MC38 mice after indicated treatments as illustrated in Figure 4A in the main text (n = 6 per group). Data (A, B) related to Figure 4B, C in the main text.

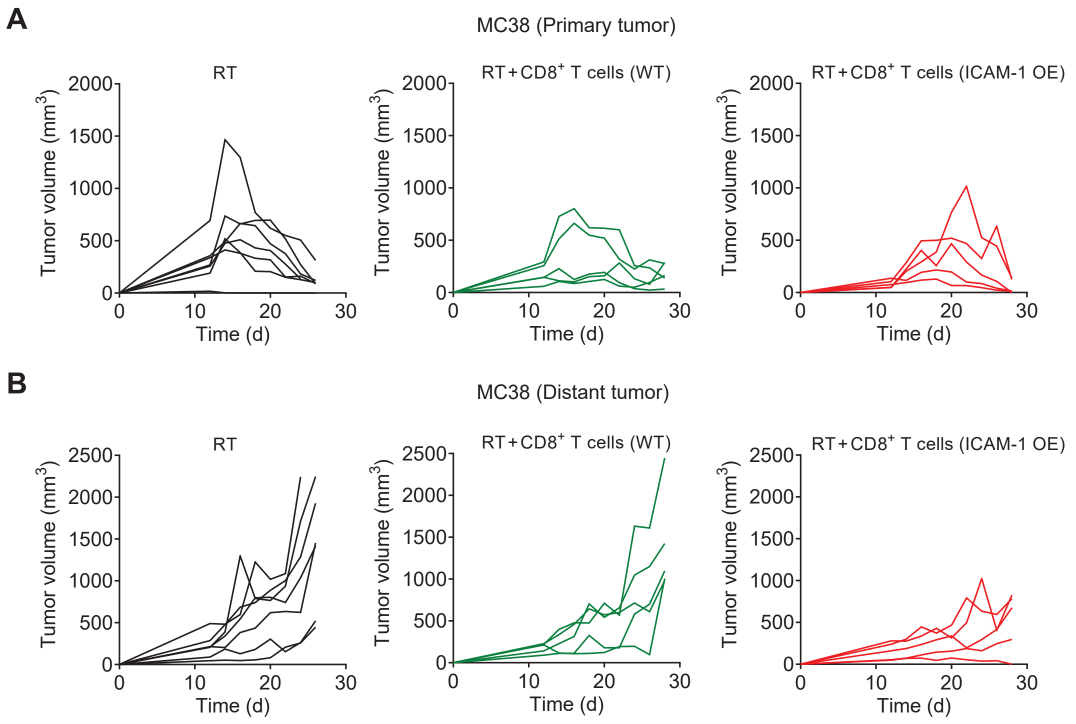

Figure S8. ICAM-1 OE augments the abscopal effect of RT in combination with ACT therapy of CD8$^+$ T cells. (A, B) Individual tumor growth curves of primary tumors (A) and distant tumors (B) in MC38 tumor-bearing mice after the indicated treatments as illustrated in Figure 4D in the main text (n = 5–7 per group). Data related to Figure 4E, F in the main text.

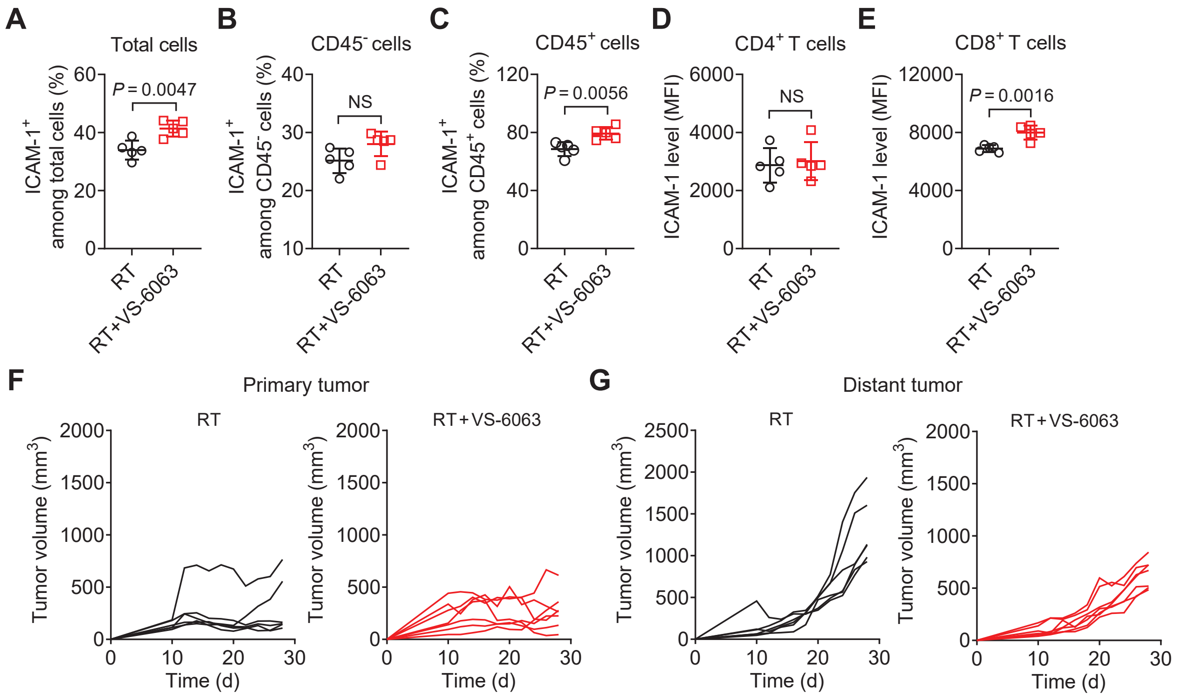

Figure S9. RT in combination with VS-6063 increases the frequency of ICAM-1$^+$ cells, ICAM-1$^+$CD45$^+$ cells, and CD8$^+$ T cells in the distant tumors. (A–E) Frequency of ICAM-1$^+$ cells among total tumor-infiltrating cells (A), CD45$^-$ cells (B), and CD45$^+$ cells (C); mean fluorescence intensity (MFI) of ICAM-1 on CD4$^+$ T cells (D) and CD8$^+$ T cells (E) in distant tumors harvested from 4T1 tumor-bearing mice after treatment with RT alone or RT plus VS-6063 using the treatment protocol as illustrated in Figure 5A in the main text (n = 5 per group). (F, G) Individual tumor growth curves of primary tumors (F) and distant tumors (G) after treatment with RT alone or RT plus VS-6063 as illustrated in Figure 5G in the main text (n = 6–7 per group). Data (F, G) related to Figure 5H, I in the main text. Data are represented as mean ± SD. *P* values were determined using unpaired Student's *t*-test (A–E); NS, not significant ($P > 0.05$).

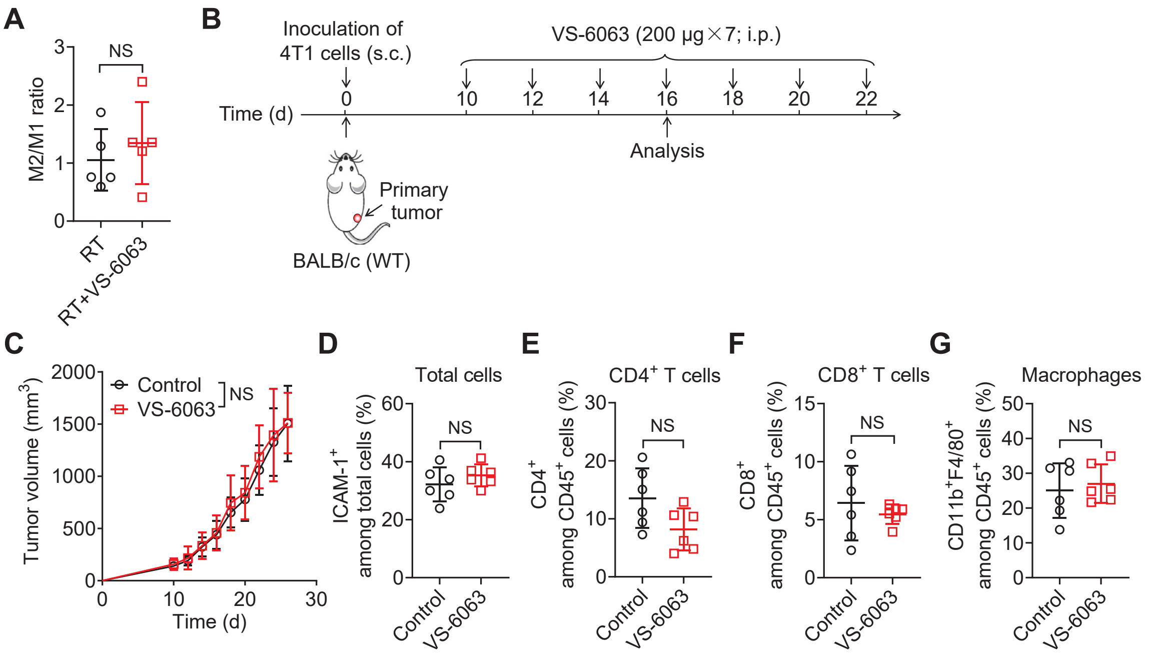

Figure S10. Treatment with VS-6063 alone does not alter the proportion of tumor-infiltrating immune cells. (A) The ratio of M2-to-M1 macrophages in the distant tumors after indicated treatments as illustrated in Figure 6A in the main text (n = 5 per group). (B) Schedule of VS-6063 treatment in the 4T1 tumor-bearing BALB/c mouse model. (C) Average tumor growth curves of the mice bearing 4T1 tumors after treatment with vehicle control or VS-6063 (n = 7 per group). (D–G) Frequencies of ICAM-1$^+$ cells among total tumor-infiltrating cells (D) and CD4$^+$ cells (E), CD8$^+$ cells (F), and macrophages (G) among CD45$^+$ cells in the 4T1 tumors after treatment with vehicle control or VS-6063 as illustrated in (B) (n = 6 per group). Data are represented as mean ± SD. *P* values were determined using unpaired Student's *t*-test (A, C–G); NS, not significant ($P > 0.05$).

Table S1. Drugs used for combination with radiotherapy in the ICAM-1-targeted imaging studies

Drug name	Source	Identifier	Drug target	Known function
cGAMP	Selleckchem	S7904	STING agonist	Activates cGAS-STING pathway and innate immune responses
SB525334	Selleckchem	S1476	TGFβR I inhibitor	Attenuates immunosuppression
Defactinib (VS-6063)	Selleckchem	S7654	FAK inhibitor	Inhibits tumor fibrosis; regulates migration of immune cells
Linrodostat (BMS986205)	Selleckchem	S8629	IDO inhibitor	Attenuates immunosuppression
IDO inhibitor 1	Selleckchem	S8557		
RRx-001	Selleckchem	S8405	G6PD inhibitor	Epigenetic regulation; CD47 downregulation; radiotherapy sensitization
Pexidartinib (PLX3397)	Selleckchem	S7818	CSF-1R inhibitor	Adjusts myeloid cell differentiation, proliferation, migration, and survival
Pomalidomide	Selleckchem	S1567	Inhibit TNF-α release	Stimulates T-cell proliferation and promotes IFN-γ and IL-2 production
Lenalidomide (CC-5013)	Selleckchem	S1029		
Maraviroc (UK-427857)	Selleckchem	S2003	CCR5 inhibitor	Inhibits regulatory T-cell differentiation and migration
Arginase inhibitor 1	MedChemExpress	HY-15775	Arginase inhibitor	Improves T_{eff} function at the tumor site
BEC HCl	Selleckchem	S7929		
BMS-1	Selleckchem	S7911	PD-1/PD-L1 inhibitor	Activates T cells
Motolimod (VTX-2337)	Selleckchem	S7161	TLR8 agonist	Activates innate immune responses
GW788388	Selleckchem	S2750	TGFβR I/II inhibitor	Attenuates immunosuppression
Eganelisib (IPI-549)	Selleckchem	S8330	PI3K inhibitor	Improves T-cell function; inhibits polarization of TAM from M0 to M2
Idelalisib	Selleckchem	S1476		
Ciforadenant (CPI-444)	Selleckchem	S6646	Adenosine A2A receptor inhibitor	Activates T cells
Fasudil (HA-1077) HCl	Selleckchem	S1573	ROCK inhibitor	Inhibits proliferation/migration of tumor cells and fibrosis
Ibrutinib (PCI-32765)	Selleckchem	S2680	BTK/ITK inhibitor	Regulates T-cell abundance and subset distribution and TCR repertoire and immune function

Note: STING, stimulator of interferon genes; cGAS, cyclic GMP–AMP synthase; TGFβR, transforming growth factor β receptor; FAK, focal adhesion kinase; IDO, indoleamine-2,3 dioxygenase; G6PD, glucose-6-phosphate dehydrogenase; CSF-1R, colony-stimulating factor-1 receptor; TNF-α, tumor necrosis factor α; IFN-γ, interferon γ; IL-2, interleukin 2; CCR5, C-C chemokine receptor type 5; T_{eff}, effector T cell; PD-

1, programmed cell death protein 1; PD-L1, programmed cell death ligand 1; TLR8, toll-like receptor 8; PI3K, phosphoinositide 3-kinase; TAM, tumor-associated macrophage; ROCK, rho kinase; BTK/ITK, Bruton's tyrosine kinase/interleukin-2-inducible T cell kinase; TCR, T-cell receptor.

Table S2. Fluorescently labeled antibodies used in this study for flow cytometric analysis.

Reagent	Source	Identifier
PE-anti-mouse CD11a	BioLegend	Clone: M17/4<br>Cat#: 101107
APC-anti-mouse CD11b	BioLegend	Clone: M1/70<br>Cat#: 101212
PE-CY7-anti-mouse CD11c	BioLegend	Clone: N418<br>Cat#: 117317
APC-anti-mouse MHCII	BioLegend	Clone: M5/114.15.2<br>Cat#: 107614
PE-anti-mouse F4/80	BioLegend	Clone: BM8<br>Cat#: 123110
APC-anti-mouse CD206	BioLegend	Clone: C068C2<br>Cat#: 141707
PE-anti-mouse CD86	BioLegend	Clone: GL-1<br>Cat#: 105007
APC-anti-mouse CD45	BioLegend	Clone: 30-F11<br>Cat#: 103112
FITC-anti-mouse CD3	BioLegend	Clone: 145-2C11<br>Cat#: 100305
PE-CY7-anti-mouse CD3	BioLegend	Clone: 500A2<br>Cat#: 152314
PerCP-anti-mouse CD8	BioLegend	Clone: 53-6.7<br>Cat#: 100731
APC-anti-mouse CD4	BioLegend	Clone: RM4-5<br>Cat#: 100516
PE-Cy7-anti-mouse CD107a	BioLegend	Clone: 1D4B<br>Cat#: 121619
APC-anti-mouse CD49	BioLegend	Clone: HMa2<br>Cat#: 103515
APC-anti-mouse PD-1	BioLegend	Clone: RMP1-30<br>Cat#: 10911
PE-anti-mouse ICAM-1	eBioscience	Clone: YN1/1.7.4<br>Cat#: 12-0541-81
FITC-anti-mouse CD8	eBioscience	Clone: 53-6.7<br>Cat#: 11-0081-82
PE-anti-mouse CD8	eBioscience	Clone: 53-6.7<br>Cat#: 12-0081-82
PerCP-Cy5.5-anti-mouse CD4	eBioscience	Clone: RM4-5<br>Cat#: 45-0042-82
AF700-anti-mouse CD45	eBioscience	Clone: 30-F11<br>Cat#: 56-0451-82
APC-anti-mouse NK1.1	eBioscience	clone PK136<br>Cat#: 17-5941-81
